# Spontaneously generated online patient experience data - how and why is it being used in health research: an umbrella scoping review

**DOI:** 10.1186/s12874-022-01610-z

**Published:** 2022-05-14

**Authors:** Julia Walsh, Christine Dwumfour, Jonathan Cave, Frances Griffiths

**Affiliations:** 1 Warwick Medical School, University of Warwick, Coventry, UK; 2 Department of Economics, University of Warwick, Coventry, UK; 3 Centre for Health Policy, University of the Witwatersrand, Johannesburg, South Africa

**Keywords:** Social media, Health research, Umbrella review, Machine learning, Natural language processing, Methods, Text analysis

## Abstract

**Purpose:**

Social media has led to fundamental changes in the way that people look for and share health related information. There is increasing interest in using this spontaneously generated patient experience data as a data source for health research. The aim was to summarise the state of the art regarding how and why SGOPE data has been used in health research. We determined the sites and platforms used as data sources, the purposes of the studies, the tools and methods being used, and any identified research gaps.

**Methods:**

A scoping umbrella review was conducted looking at review papers from 2015 to Jan 2021 that studied the use of SGOPE data for health research. Using keyword searches we identified 1759 papers from which we included 58 relevant studies in our review.

**Results:**

Data was used from many individual general or health specific platforms, although Twitter was the most widely used data source. The most frequent purposes were surveillance based, tracking infectious disease, adverse event identification and mental health triaging. Despite the developments in machine learning the reviews included lots of small qualitative studies. Most NLP used supervised methods for sentiment analysis and classification. Very early days, methods need development. Methods not being explained. Disciplinary differences - accuracy tweaks vs application. There is little evidence of any work that either compares the results in both methods on the same data set or brings the ideas together.

**Conclusion:**

Tools, methods, and techniques are still at an early stage of development, but strong consensus exists that this data source will become very important to patient centred health research.

**Supplementary Information:**

The online version contains supplementary material available at 10.1186/s12874-022-01610-z.

## Background

The rapid growth of social media (SM) has led to fundamental changes in the way that people look for and share health related information. Of a global population of 7.6 billion, almost half (3.7 billion) are classified as active (over once a month) social media users [1], with 72% of US adults using it for health purposes [2], either as a first or second-line health information source [3] or exchange resource [4, 5]. Restrictions and local lockdowns due to the global COVID-19 pandemic are likely to have led to an even greater use of health-related online use, as individuals may have avoided personal visits to clinicians or been unable to access treatments [6]. Posts written by individuals on social media platforms are creating vast resources of spontaneously generated online patient experience (SGOPE) data in the form of unstructured text.

As the numbers of individuals using the internet for health-related purposes continues to rise, there has been a corresponding increased interest in exploring this online user generated content as a data source for health research [7]. The potential benefits of social media as a research resource for healthcare include reducing research costs [8], improving patient empowerment [9], engagement [10] and health communication [11]. Despite the methodological complexities of analysing large volumes of unstructured natural language text there has been increased interest from both commercial and academic researchers into methods of generating knowledge from it, and new methods are developing rapidly [12–14]. Although the use of health-related social media as a data source is a relatively new subject area, it is being actively researched across many other disciplines, including computer science, sociology, philosophy, and business. The volume of published literature is growing rapidly and includes both academic and grey sources, but as yet there is little literature bringing together the developments in the area [15, 16]. A review of reviews from 2018 looking at the potential uses, benefits, harms and tools was inconclusive in terms of the effectiveness and uses of SM as a data source for mental health research, concluding that better research designs were needed [17]. As far as we are aware, this is the first review of this type since then. It summarises the current state of the art of how and why SGOPE data is being utilised in health research by conducting a scoping umbrella review of the recent literature with a particular focus on SGOPE data.

### Aims & objectives

This review examines how SGOPE data is currently being used within health research. Our main research question for this review is “How and for what purposes is SGOPE data currently being utilised within health research?”. Sub-questions include:

- Which sites / platforms are being used as data sources?
- What purposes is SGOPE data used for?
- What tools and methods are being used in the studies?
- What are the knowledge gaps and areas of future research needed?

## Methods

### Study design, reason & justification

This study is an umbrella scoping review. An umbrella review is a form of knowledge synthesis which by summarising existing review papers aims to describe the subject area, what is currently known about it and identify the gaps in knowledge [18]. Scoping reviews are particularly useful when looking to get a broad overview of an emerging area, drawing together the key concepts and what it encompasses [19]. We chose this combined novel method for two main reasons. Firstly, comparing existing reviews gives a wide overview of the subject area, highlighting existing evidence and illustrating how researchers across the various disciplines are exploring the topic. By avoiding the repetition of searches, screening of individual papers, and the resynthesizing of existing studies it provides an overall picture of the current state of the art that can be used as a broad base to build from [18]. Secondly, as the literature base is so varied, a scoping review enables the inclusion of any other relevant review literature that would not otherwise be included within a systematic review, such as grey or opinion literature. Much of the most current research around natural language processing (NLP) is interdisciplinary rather than being published solely in the mainstream health based journals [20]. Although not always subject to the peer review process of the more traditional journals, these other sources can be an important source of information for a review on such a rapidly evolving subject area. Widening the scope adds both to the depth and breadth of the literature as well as reducing the potential for any publication bias. Searching across disciplines, especially in an area where the terminology varies and evolves rapidly means that it is difficult to use tightly defined search terms. Many relevant keywords relating to SM have yet to be indexed into the MeSH system so it was not possible to rely purely on MeSH terms for searching [7, 21]. Scoping review methods do not require a formal critical appraisal of the literature [19]. We conducted this umbrella review following the methodology suggested by the Joanna Briggs Institute [18].

### Search strategy

We searched the following databases; Medline, Embase, PubMed, PsychInfo, Web of Science, ACM and IEEE Xplore. We also searched Google Scholar, Twitter, Google and other text or opinion literature. Additional literature, both published and ‘grey’, such as conference reports, was added from reference lists and an existing bibliography previously compiled by JW. Final searches were conducted on January 21st, 2021.

### Search terms

**Fig. 1:**
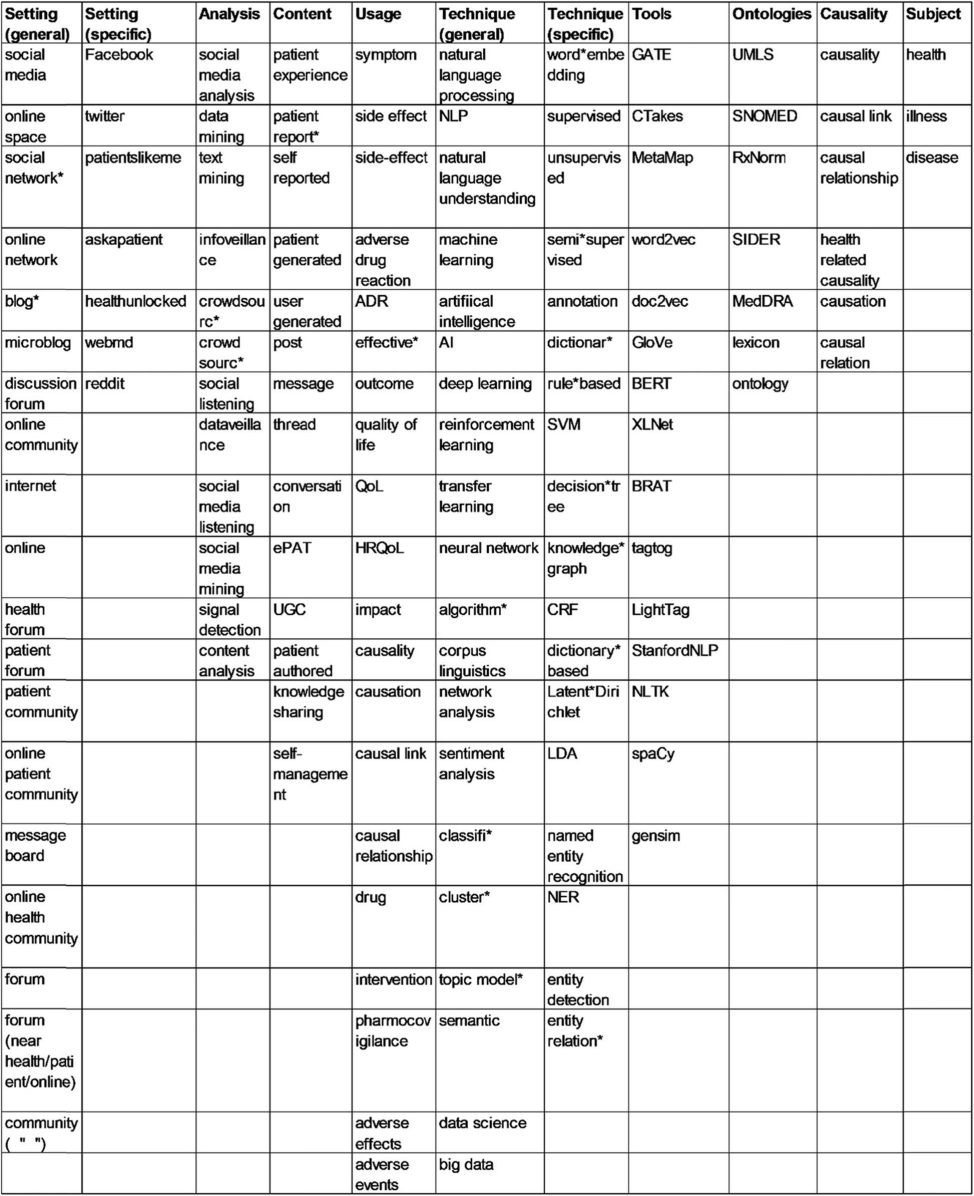
Search term clusters

The original search terms were based on keywords, first based on input from a research librarian, then agreed by JW and CD, clustered around the main areas of setting, analysis, content, usage, and methods (Fig. 1). We combined the keywords in various options and then conducted the searches as an iterative process, repeated as further search terms were identified to optimise the efficiency and targeting of the search process. Wildcards (*) were used where possible to pick up multiple word endings or ambiguities over hyphen usage.

### Inclusion/ exclusion criteria

Papers were included if they were any type of review; systematic, scoping, literature, or general that included analysis of SGOPE data for health research. Non review papers, those not referring to SGOPE usage, not health related, entirely mathematical, or statistical, not in English or published before 2015 were excluded.

### Study selection

After duplicate removal, the initial screening was a three phased approach by two reviewers (JW and CD). Using the Rayyan tool [22] to assess the reviews, the remaining papers were initially classified independently as include, unsure or exclude based on the title or headline. Both JW and CD then read the abstracts or first paragraph of those not excluded. Full texts were retrieved of relevant papers. Initial agreement rate was 86%. Results were compared and disagreements resolved through discussion while comparing perspectives of the inclusion and exclusion criteria of the full text of the papers until agreement was reached. Although critical appraisal is not required for a scoping review, the reviews were informally assessed using the questions from the Confidence in the Evidence from Reviews of Qualitative research (CERQual) appraisal tool (Table 1) to ensure their suitability for inclusion.

**Table 1:** Components of CERQual appraisal tool (GRADE CERQual, 2017)

Methodological limitations	Are the methods suitable for this project?
Relevance	Do the findings relate to the research question?
Coherence	How well does the data relate to the finding?
Adequacy	Richness & quantity of data supporting the finding

The final selection of included papers was collated into a marked list on the Web of Science database for basic bibliometric analysis.

### Data extraction/ analysis

The data extraction form was designed by JW and FG. The extracted data from each included paper comprised title, author(s), date of publication, journal, keywords, review type, objectives, research questions (where stated), number and type of included studies, settings and population studied, data sources, date ranges of included studies, key findings, future research needed if identified, and strengths and limitations if included. This enabled us to analyse the reviews in line with the research questions. Frequency analysis was performed on the author generated words for each included paper, and a word cloud generated.

## Results

**Fig. 2:**
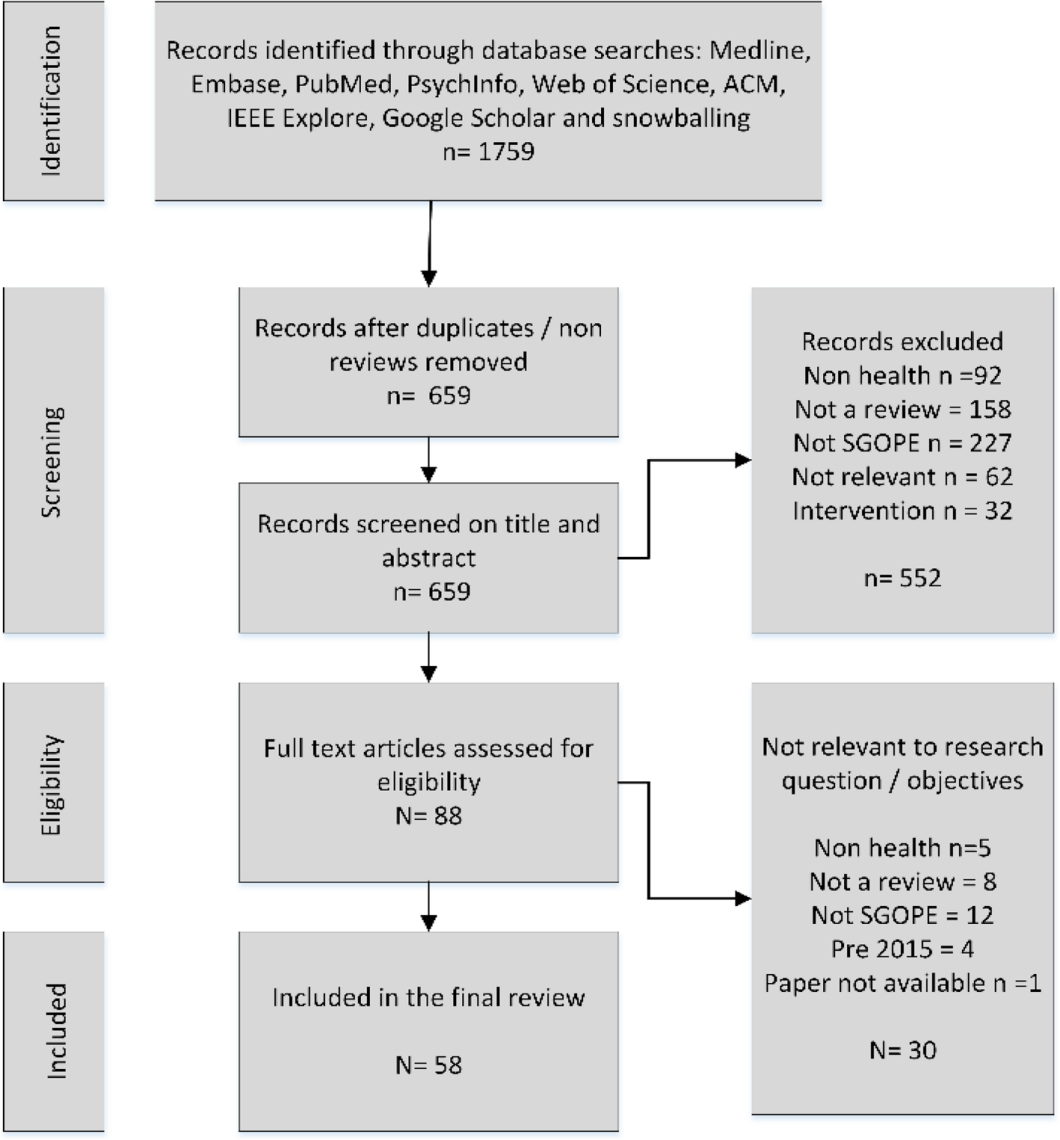
Prisma flow diagram

Of the 1759 records initially identified from the searches, 58 were included in the final review. Details and characteristics of the included papers are summarised in Table 2. The PRISMA flow diagram (Fig. 2) shows the number of papers included and excluded at each stage of the process.

**Table 2:** Characteristics of included review papers

Ref	Title	Review Aims/Objectives	Area	Data Source	Review type	No Papers
Abbe 2016 [23]	Text mining applications in psychiatry: a systematic literature review	Two specific objectives: (1) to collect and analyse applications from the studies reviewed to assess the benefits and limitations of using TM; and (2) to identify new opportunities for use of TM in psychiatry.	Mental health	Online posts, qual studies, EHRs, biomed literature	Systematic	38
Abd Rahman 2020 [24]	Application of Machine Learning Methods in Mental Health Detection: A Systematic Review	The main purpose of this paper is to explore the adequacy, challenges, and limitations of a mental health problem detection based on OSNs data. The objective of this systematic literature review is to conduct a critical assessment analysis on detection of mental health problems using OSNs. We also investigated the appropriateness of this pre-mental health detection by identifying its data analysis method, comparison, challenges, and limitations.	Mental Health	Mostly Twitter or Sina Weibo (Chinese Twitter)	Systematic	22
Al-Garadi 2016 [25]	Using online social networks to track a pandemic: A systematic review	This study aims to investigate the adequacy and limitations of pandemic surveillances based on OSN data.	Infectious disease	Mostly Twitter	Systematic	20
Allen 2016 [26]	Long-Term Condition Self-Management Support in Online Communities: A Meta-Synthesis of Qualitative Papers	To understand the negotiation of long-term condition illness-work in patient online communities and how such work may assist the self-management of long-term conditions in daily life.	Chronic	Mostly disease specific / Gen health sites / FB	Systematic	21
Barros 2020 [27]	The Application of Internet-Based Sources for Public Health Surveillance (Infoveillance): Systematic Review	aimed to assess research findings regarding the application of IBSs for public health surveillance (infodemiology or infoveillance).	Public Health	SM, search queries	Systematic	162
Calvo 2017 [20]	Natural language processing in mental health applications using non-clinical texts	To highlight areas of research where NLP has been applied in the mental health literature and to help develop a common language that draws together the fields of mental health, human-computer interaction, and NLP.	Mental health	Mostly Twitter	Scoping	23
CastiiloSanchez 2020 [28]	Suicide Risk Assessment Using Machine Learning and Social Networks: A Scoping Review	Aims to identify the machine learning techniques used to predict suicide risk based on information posted on social networks.	Mental Health	any but mostly Twitter	Scoping	16
Charles-Smith 2015 [29]	Using Social Media for Actionable Disease Surveillance and Outbreak Management: A Systematic Literature Review	1. Q1. Can social media be integrated into disease surveillance practice and outbreak management to support and improve public health? 2. Q2. Can social media be used to effectively target populations, specifically vulnerable populations, to test an intervention and interact with a community to improve health outcomes?	Infectious disease	Mostly Twitter (81%)	Systematic	33
Cheerkoot-Jalim 2020 [30]	A systematic review of text mining approaches applied to various application areas in the biomedical domain	To identify the different text mining approaches used in different application areas of the biomedical domain, the common tools used, and the challenges of biomedical text mining as compared to generic text mining algorithms.	Any	EHR, Biomed literature, SM	Systematic	34
Convertino 2018 [31]	The usefulness of listening social media for pharmacovigilance purposes: a systematic review	To evaluate the usefulness and quality of signals from social media listening.	ADR	Varied	Systematic	38
Demner-Fushman 2016 [32]	Aspiring to Unintended Consequences of Natural Language Processing: A Review of Recent Developments in Clinical and Consumer-Generated Text Processing	To review work over the past two years in Natural Language Processing (NLP) applied to clinical and consumer-generated texts	Any	Clinical & UG texts.	General review	NS
Dobrossy 2020 [33]	“Clicks, likes, shares and comments” a systematic review of breast cancer screening discourse in social media	we had two aims: first, to assess the volume, participants, and content of breast screening social media communication and second, to find out whether social media can be used by screening organisers as a channel of patient education.	Breast Cancer	any but mostly Twitter	Systematic	17
Dol 2019 [34]	Health Researchers’ Use of Social Media: Scoping Review	To explore how social media is used by health researchers professionally, as reported in the literature	Any	Varied	Scoping	414
Dreisbach 2019 [35]	A systematic review of natural language processing and text mining of symptoms from electronic patient-authored text data	To synthesize the literature on the use of natural language processing (NLP) and text mining as they apply to symptom extraction and processing in electronic patient-authored text (ePAT)	Symptoms	Varied	Systematic	21
Drewniak 2020 [36]	Risks and Benefits of Web-Based Patient Narratives: Systematic Review	This review aimed to evaluate whether research-generated Web-based patient narratives have quantifiable risks or benefits for (potential) patients, relatives, or health care professionals	Any	Any SM	Systematic	17
Edo-Osagie 2020 [37]	A scoping review of the use of Twitter for public health research	Aims to review and synthesize the literature on Twitter applications for public health, highlighting current research and products in practice.	Any	Twitter	Scoping	92
Falisi 2017 [38]	Social media for breast cancer survivors: a literature review	To provide a systematic synthesis of the current literature in order to inform cancer health communication practice and cancer survivorship research.	Breast cancer	Online support groups	Systematic	98
Filannino 2018 [39]	Advancing the State of the Art in Clinical Natural Language Processing through Shared Tasks	To review the latest scientific challenges organized in clinical Natural Language Processing (NLP) by highlighting the tasks, the most effective methodologies used, the data, and the sharing strategies.	Any	Twitter/ReachOut forum	General review	17
Fung 2016 [40]	Ebola virus disease and social media: A systematic review	Ebola virus disease and social media, especially to identify the research questions and the methods used to collect and analyse social media	Infectious disease	Mostly Twitter & YouTube	Systematic	12
Gianfredi 2018 [41]	Harnessing Big Data for Communicable Tropical and Sub-Tropical Disorders: Implications from a Systematic Review of the Literature	To systematically assess the feasibility of exploiting novel data streams (NDS) for surveillance purposes and/or their potential for capturing public reaction to epidemic outbreaks.	Infectious disease	Varied but mostly Twitter	Systematic	47
Giuntini 2020 [42]	A review on recognizing depression in social networks: challenges and opportunities	investigates the state-of-the-art of how sentiment and emotion analysis approaches can identify depressive disorders in social networks.	Mental Health	Any: mostly Twitter	Systematic	26
Gohil 2018 [15]	Sentiment Analysis of Health Care Tweets: Review of the Methods Used	To review the methods used to measure sentiment for Twitter-based health care studies.	Any	Twitter	Systematic	12
Golder 2015 [43]	Systematic review on the prevalence, frequency, and comparative value of adverse events data in social media	To summarize prevalence, frequency, and comparative value of information on the adverse events of healthcare interventions from user comments and videos in social media.	ADR	Mostly discussion forums	Systematic	51
Gonzalez-Hernandez 2017 [44]	Capturing the Patient’s Perspective: a Review of Advances in Natural Language Processing of Health-Related Text	To review the recently published literature discussing the application of NLP techniques for mining health-related information from EHRs and social media posts. To provide a scope of the trends and advances in capturing the patient’s perspective on health within the last three years.	Any	SM & EHRs	General review	87
Gupta 2020 [45]	Social media-based surveillance systems for healthcare using machine learning: A systematic review	We review the recent work, trends, and machine learning (ML) text classification approaches used by surveillance systems seeking social media data in the healthcare domain. We also highlight the limitations and challenges followed by possible future directions that can be taken further in this domain.	Any	Twitter 64%	Systematic	26
Hamad 2016 [46]	Toward a Mixed-Methods Research Approach to Content Analysis in The Digital Age: The Combined Content-Analysis Model and its Applications to Health Care Twitter Feeds	To identify studies on health care and social media that used Twitter feeds as a primary data source and CA as an analysis technique.	ADR	Twitter	Narrative review	18
Ho 2016 [75]	Data-driven Approach to Detect and Predict Adverse Drug Reactions	Compares omics, social media and EHRs as sources of ADR knowledge	ADR	Any SM	General review	22
Injadat 2016 [47]	Data mining techniques in social media: A survey	Techniques, areas, performance, comparison of techniques, strengths and weaknesses of data mining methods	Any	Any SM	Survey	66
Karmegan 2020 [48]	A Systematic Review of Techniques Employed for Determining Mental Health Using Social Media in Psychological Surveillance During Disasters	Our review aims to analyse the possibility, effectiveness, and procedures of using social media data to understand the emotional and psychological impact of an unforeseen disaster on the community.	Mental Health	Any SM: mostly Twitter	Systematic	18
Kim 2017 [49]	Scaling Up Research on Drug Abuse and Addiction Through Social Media Big Data	To determine how social media big data can be used to understand communication and behavioural patterns of problematic use of prescription drugs.	Substance misuse	Twitter	Critical	8
Lafferty 2015 [50]	Perspectives on social media in and as research: A synthetic review	To summarize findings, opinions and discussion about the use of SoMe in research, including examples from psychiatry.	Mental health	Varied	Systematic	56
Lardon 2015 [51]	Adverse Drug Reaction Identification and Extraction in Social Media: A Scoping Review	To explore the breadth of evidence about the use of social media as a new source of knowledge for pharmacovigilance.	ADR	Mainly online forums +Twitter/blogs	Scoping	24
Lau 2019 [52]	Artificial Intelligence in Health: New Opportunities, Challenges, and Practical Implications	To summarise the state of the art during the year 2018 in consumer health informatics	Any	Any SM	General review	14
Lopez-Castroman 2019 [53]	Mining social networks to improve suicide prevention: A scoping review	Narrative review of possible suicidal behaviours on social networks	Mental health	NS	Scoping	NS
Mavragani 2020 [54]	Infodemiology and Infoveillance: Scoping Review	The aim of this paper is to provide a scoping review of the state-of-the-art in infodemiology along with the background and history of the concept, to identify sources and health categories and topics, to elaborate on the validity of the employed methods, and to discuss the gaps identified in current research.	Any	Mostly Twitter	Scoping	338
Neveol 2017 [55]	Making Sense of Big Textual Data for Health Care: Findings from the Section on Clinical Natural Language Processing	To identify the best clinical NLP papers of 2016	Any	SM + EHRs	General review	5
Neveol 2018 [21]	Expanding the Diversity of Texts and Applications: Findings from the Section on Clinical Natural Language Processing of the International Medical Informatics Association Yearbook	Summarize recent research / best papers for clinical NLP in 2017	Any	Any SM	General review	15
Patel 2015 [56]	Social Media Use in Chronic Disease: A Systematic Review and Novel Taxonomy	To evaluate clinical outcomes from applications of contemporary social media in chronic disease; to develop a conceptual taxonomy to categorize, summarize, and then analyse the current evidence base; and to suggest a framework for future studies on this topic	Chronic	Any SM	Systematic	42
Pourebrahim 2020 [57]	Adverse Drug Reaction Detection Using Data Mining Techniques: A Review Article	The aim of this study is to study, review and challenge the methods of ADR diagnosis by data mining on social media, especially Twitter.	ADR	Any SM: mostly Twitter	General	0
Qiao 2020 [58]	A Systematic Review of Machine Learning Approaches for Mental Disorder Prediction on Social Media	The purpose of this paper is to provide a systematic overview of SM studies in the mental disorder detection field.	Mental Health	Facebook, Twitter, Reddit, Tumblr, Instagram	General	0
Ru & Yao 2019 [7]	A Literature Review of Social Media-Based Data Mining for Health Outcomes Research	To summarize key points of the area in data accessibility, textual data pre-processing methods, analysis methods, opportunities, and challenges.	Any	Any SM	General review	19
Santos 2019 [59]	Datamining and machine learning techniques applied to public health problems: A bibliometric analysis from 2009 to 2018	To: (i) analyse the number of papers published from 2009 to 2018 (10 years) due to the increasing number of publications and dissemination of ML in public health; (ii) identify the journals with the greatest number of papers; (iii) determine which techniques, programming languages and software tools are most widely used in the field of DM applied to public health; (iv) identify which countries and databases were targeted by these studies; (v) analyse which public health classes were tackled by these papers and (vi)identify which papers were most frequently cited in the literature.	Public health	Any SM	Bibliometric	250
Sarker 2019 [60]	Mining social media for prescription medication abuse monitoring: a review and proposal for a data-centric framework	To present a methodological review of social media-based PM abuse or misuse monitoring studies, and to propose a potential generalizable, data-centric processing pipeline for the curation of data from this resource.	Substance misuse	Twitter / Facebook / Reddit	General review	39
Sharma 2016 [61]	Identifying Complementary and Alternative Medicine Usage Information from Internet Resources. A Systematic Review	Identify and highlight research issues and methods used in studying Complementary and Alternative Medicine (CAM) information needs, access, and exchange over the Internet.	CAM	Any SM	Systematic	120
Sharma 2020 [62]	Sentiment analysis of social media posts on pharmacotherapy: A Scoping Review	The aim of this scoping review was to describe the available evidence as it pertains to SA of Social Media specifically about pharmacotherapy. Themes will be generated about the published uses of SA and the real-world implications of the knowledge generated.	Any	Any SM: mostly Twitter	Scoping	10
Sinnenberg 2017 [63]	Twitter as a Tool for Health Research: A Systematic Review	To systematically review the use of Twitter in health research, define a taxonomy to describe Twitter use, and characterize the current state of Twitter in health research.	Health research	Twitter	Systematic	137
Skaik 2020 [64]	Using Social Media for Mental Health Surveillance: A Review	This systematic review aims to analyse the literature on using social media posts to predict mental disorders using ML and NLP methods that could be useful for mental health surveillance and presents the cutting-edge techniques in predictive analysis of suicide ideation and depression at the population-level. It also points at the gaps that need further research from the perspective of the data, the models, and evaluation procedures.	Mental Health	Any SM	General	110
Staccini 2017 [65]	Secondary Use of Recorded or Self-expressed Personal Data: Consumer Health Informatics and Education in the Era of Social Media and Health Apps	To summarize the state of the art during the year 2016 in the areas related to consumer health informatics and education with a special emphasis in secondary use of patient data.	Any	Any SM	Systematic	5
Su 2020 [66]	Deep learning in mental health outcome research: a scoping review	The goal of this study is to review existing research on applications of DL algorithms in mental health outcome research.	Mental Health	SM, EHR, etc	Scoping	57
Tricco 2018 [67]	Utility of social media and crowd-intelligence data for pharmacovigilance: a scoping review	Review the literature regarding using SM conversations for ADR detection	ADR	Any SM	Scoping	70
Vilar 2018 [68]	Detection of drug-drug interactions through data mining studies using clinical sources, scientific literature, and social media	To review datamining as a method of detecting drug-drug interactions	ADR	SM/ EHRs. FAERS, WHO	General review	NS
Wilson 2015 [69]	Using blogs as a qualitative health research tool: A scoping review	To identify how blogs are being used in health research to date and whether blogging has potential as a useful qualitative tool for data collection. Our purpose was to summarize the extent, range, and nature of research activity using blogs.	Any	blogs	Scoping	44
Wong 2018 [70]	Natural Language Processing and Its Implications for the Future of Medication Safety: A Narrative Review of Recent Advances and Challenges	To review methods of identifying adverse events from free text	ADR	SM + EHRs	General review	12
Wongkoblap 2017 [71]	Researching Mental Health Disorders in the Era of Social Media: Systematic Review	To explore the scope and limits of cutting-edge techniques that researchers are using for predictive analytics in mental health and to review associated issues, such as ethical concerns, in this area of research.	Mental health	Various SM	Systematic	48
Yin 2019 [16]	A systematic literature review of machine learning in online personal health data	To systematically review the effectiveness of applying machine learning (ML) methodologies to UGC for personal health investigations.	Any	Any SM: mostly Twitter	Systematic	103
Zhang 2018 [72]	Using Twitter for Data Collection with Health-Care Consumers: A Scoping Review	To provide an overview of previously published literature describing Twitter as a data collection method with health-care consumers and provide researchers with considerations when potentially using this data collection approach.	Any	Twitter	Scoping	17
Zhang 2020 [73]	When Public Health Research Meets Social Media: Knowledge Mapping From 2000 to 2018	Aims to examine research themes, the role of social media, and research methods in social media–based public health research published from 2000 to 2018	Any	Any SM	Review	3419
Zunic 2020 [74]	Sentiment Analysis in Health and Well-Being: Systematic Review	This study aimed to establish the state of the art in SA related to health and well-being by conducting a systematic review of the recent literature. To capture the perspective of those individuals whose health and well-being are affected, we focused specifically on spontaneously generated content and not necessarily that of health care professionals.	Any	Various SM	Systematic	86

### General characteristics

**Fig. 3:**
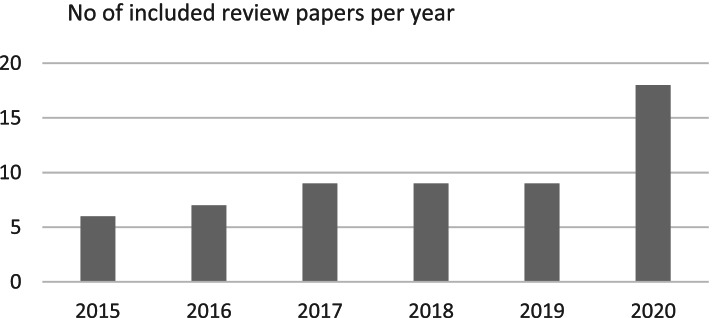
Included review papers per year

The 58 included reviews covered the period 2015 to 2020 with the reported number of associated papers published increasing each year, especially since 2017 [27, 34, 54, 59, 63, 73]. This is illustrated in the breakdown of included review papers by publication year (Fig. 3).

The included studies came from wide range of journals, although only six journals provided more than one review. Journal of Internet Mediated Research (JIMR) contributed 11/58 (19%), Yearbook of Medical Informatics 7/58 (12%), and IEEE 3/58 (5%), while PloSone, Journal of Biomedical Informatics and the International Journal of Qualitative Methods each provided 2/58 (3%). The other 31 papers originated from individual journals from a wide range of research areas (Table 3).

**Table 3:** Included papers by journal

Review	Journal
Allen 2016 [26]	Journal of Medical Internet Research
Barros 2020 [27]	Journal of Medical Internet Research
Dol 2019 [34]	Journal of Medical Internet Research
Drewniak 2020 [36]	Journal of Medical Internet Research
Hamad 2016 [46]	Journal of Medical Internet Research
Kim 2017 [49]	Journal of Medical Internet Research
Mavragani 2020 [54]	Journal of Medical Internet Research
Zhang 2020 [73]	Journal of Medical Internet Research
Lardon 2015 [51]	Journal of Medical Internet Research
Wongkoblap 2017 [71]	Journal of Medical Internet Research
Lopez-Castroman 2019 [53]	Journal of Medical Internet Research
Demner-Fushman 2016 [32]	Yearbook of Medical Informatics
Filannino 2018 [39]	Yearbook of Medical Informatics
Gonzalez-Hernandez 2017 [44]	Yearbook of Medical Informatics
Lau 2019 [52]	Yearbook of Medical Informatics
Neveol 2017 [55]	Yearbook of Medical Informatics
Neveol 2018 [21]	Yearbook of Medical Informatics
Staccini 2017 [65]	Yearbook of Medical Informatics
Pourebrahim 2020 [57]	IEEE
Qiao 2020 [58]	IEEE
Abd Rahman 2020 [24]	IEEE
Dobrossy 2020 [33]	PLoSone
Charles-Smith 2015 [29]	PLoSone
Wilson 2015 [69]	International Journal of Qualitative Methods
Zhang 2018 [72]	International Journal of Qualitative Methods
Al-Garadi 2016 [25]	Journal of Biomedical Informatics
Gupta 2020 [45]	Journal of Biomedical Informatics
Skaik 2020 [64]	ACM Computer Survey
Fung 2016 [40]	American Journal of Infection Control
Sinnenberg 2017 [63]	American Journal of Public Health
Patel 2015 [56]	American Journal of Medicine
Tricco 2018 [67]	BMC Medical Informatics & Decision Making
Golder 2015 [43]	British Journal of Clinical Pharmacology
Vilar 2018 [68]	Briefings in Bioinformatics
Edo-Osagie 2020 [37]	Computers in Biology & Medicine
Santos 2019 [59]	Computers & Industrial Engineering
Ho 2016 [75]	Current Pharmaceutical Design
Karmegan 2020 [48]	Disaster medicine and public health preparedness
Convertino 2018Kar [31]	Expert Opinion on Drug Safety
Gianfredi 2018 [41]	Frontiers in Public Health
Dreisbach 2019 [35]	International Journal of Medical Informatics
Lafferty 2015 [50]	International Review of Psychiatry
Abbe 2016 [23]	International Journal of Methods in Psychiatric Research
Sarker 2019 [60]	Journal of American Medical Informatics
Falisi 2017 [38]	Journal of Cancer Survivorship
Castiilo-Sanchez 2020 [28]	Journal of Medical Systems
Cheerkoot-Jalim 2020 [30]	Journal of Knowledge Management
Yin 2019 [16]	JAMA Medical Informatics
Zunic 2020 [74]	JMIR Medical Informatics
Gohil 2018 [15]	JMIR Public Health Surveillance
Giuntini 2020 [42]	Journal of Ambient Intelligence and Humanized Computing
Sharma 2016 [61]	Methods of Information in Medicine
Calvo 2017 [20]	Natural Language Engineering
Injadat 2016 [47]	Neurocomputing
Sharma 2020 [62]	Pharmacology Research and Perspectives
Wong 2018 [70]	Pharmocotherapy
Ru & Yao 2019 [7]	Social Web and Health Research (book)
Su 2020 [66]	Translational Psychiatry

**Fig. 4:**
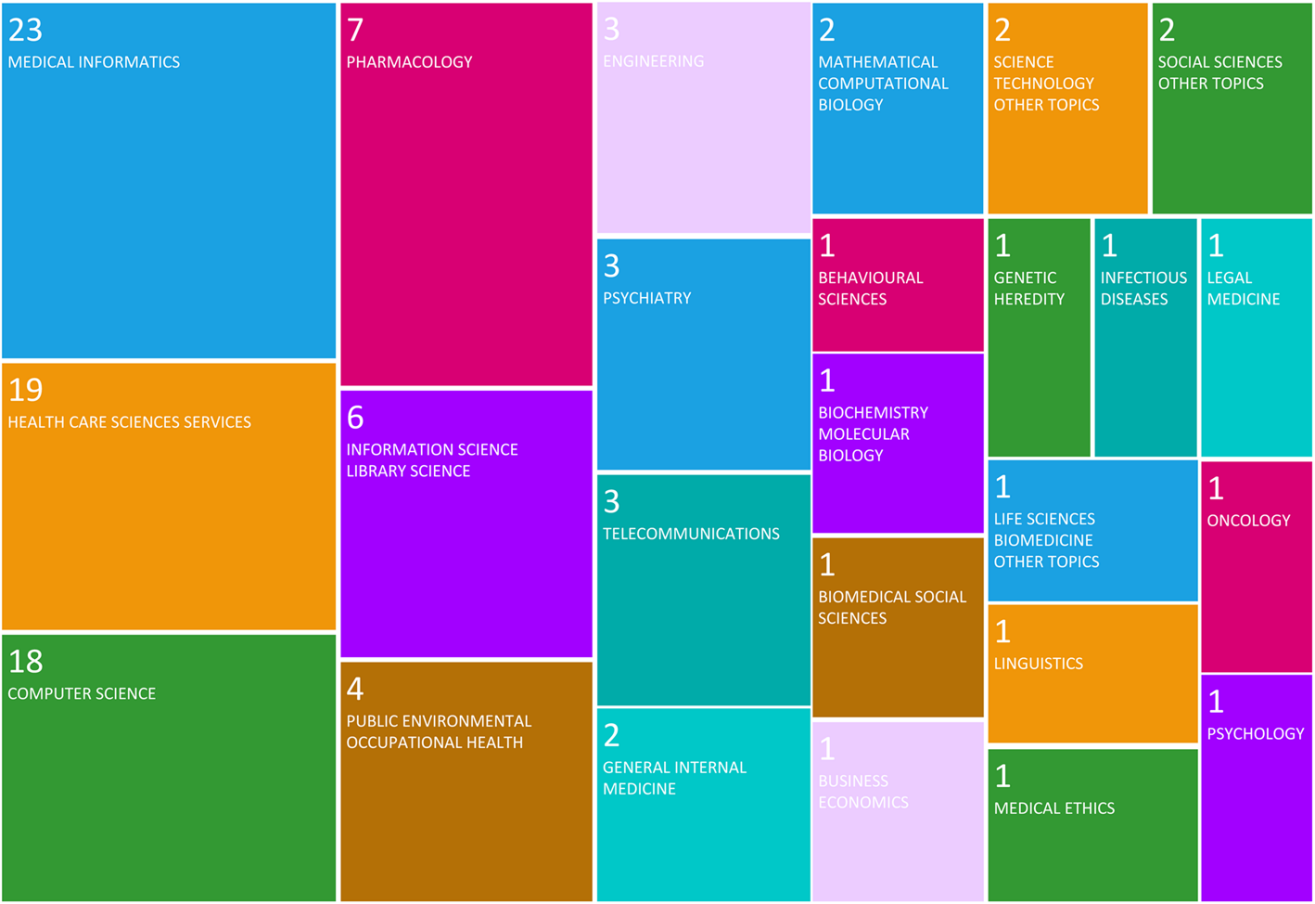
Research area of included reviews – WoS bibliometric analysis (WoS 2020)

The interdisciplinary nature of the topic is reflected in the included tree map of the research areas as defined by the Web of Science database bibliometric analysis that the papers are from (Fig. 4). Furthermore, one of the larger reviews (414 papers) analysed the discipline of each of the first authors; finding that in 90 papers (22%) they were either from a non-health or unspecified background [34].

The characteristics of each review: the aims, health condition of interest, data sources, review type and number of included papers of the 58 included papers are shown in Table 2. In terms of the methodology of the included reviews, almost half (27/58), were described as systematic, with 15/58 being general, 12/58 scoping, with one each of narrative, critical, survey and bibliometric. The number of studies in the included reviews ranged from 5 [55, 65] to 3419 [73], with an average of 118.

In line with the inclusion criteria the included reviews cover two main areas; how spontaneously generated data is used within health research and the methods and tools that are used to analyse it. Just over half (34/58) of the reviews cover both questions while 13/58 were primarily focused on uses and 11/58 mainly focused on the methods used (Table 4).

**Table 4:** Categorisation of the main purpose of the review

Uses	[22, 29, 32, 36, 38, 45, 50, 55, 64, 66, 69, 74, 75]
Methods	[20, 23, 24, 37, 46, 49, 65, 70, 72, 73]
Both	[7, 19, 23–27, 35, 37–40, 42, 44, 45, 47–49, 51–53, 55, 59, 60, 62–64, 67, 68, 71–73, 75, 76]

**Fig. 5:**
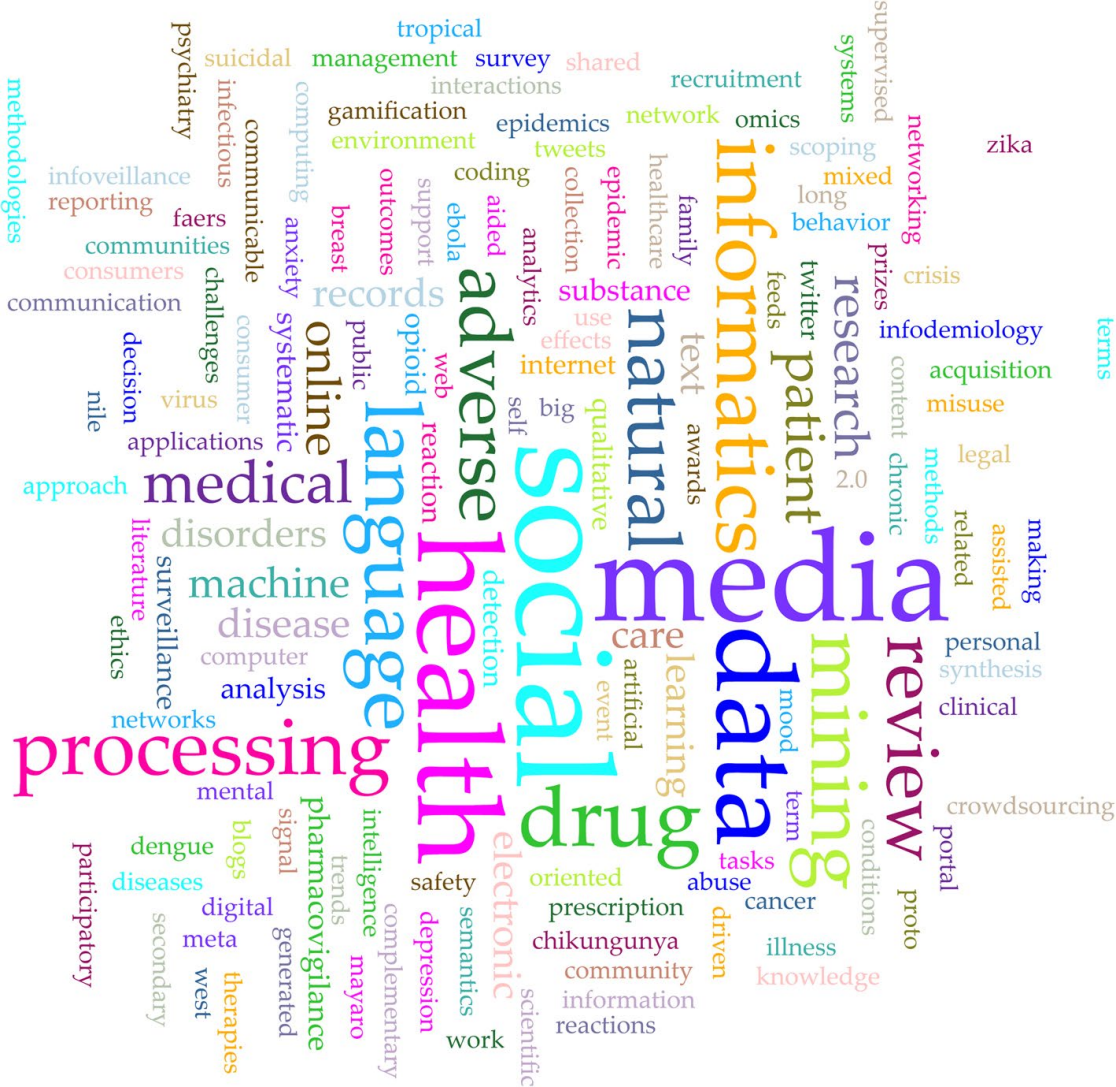
Word cloud of author generated keywords

The word cloud of the individual author generated keywords illustrates the range and frequency of the intended purposes of the included reviews (Fig. 5).

### RQ1: which SGOPE sites and platforms are used as data sources?

Twitter has been by far the most utilised data source, although a wide variety of general social networks and disease specific communities have also been used. Six (10%) of the reviews looked entirely at Twitter based studies [15, 37, 46, 49, 63, 72]. A further eighteen (31%) reviews included a wider range of sites but reported that Twitter was the most frequently used source [20, 24, 25, 28, 29, 33, 39–41, 45, 48, 54, 59, 60, 62, 67, 74]. Both general health sites and disease specific communities covering a wide range of conditions were also widely accessed [20, 23, 24, 26, 31, 33, 35, 42, 45, 51, 58, 74]. One review focused entirely on looking at the potential of blogs as a qualitative data source [69]. Six (10%) reviews used both SGOPE and electronic health records (EHRs) [30, 32, 44, 55, 66, 70] while Abbe [23] used a combination of online posts, EHR’s, biomedical literature and qualitative studies.

Four reviews highlighted that although there were exceptions, most of the individual papers within the reviews used data from a single source [20, 41, 43, 67].

### RQ2: what purposes is SGOPE data used for?

The identified use cases for SGOPE data extended from improving public health at a population level to fine grained understanding of patient perspectives. We summarised the varied aims, outcomes and key findings from the included reviews in Table 5.

**Table 5:** Aims, outcomes, key findings methods used and future research suggestions

Ref	Paraphrased Aims	Area	Outcomes Assessed	Key Findings Paraphrased	Methods Mentioned	Future Research
Abbe 2016 [23]	Benefits & limitations. Current and potential uses in psych.	Mental health	Objectives of studies, and topic modelling methods /tools used for pre-processing and analysis.	Identified four main areas of application: Psychopathology, patient perspective, medical records, medical literature. A data source that cannot be ignored. Techniques and topics heterogenous. Basic capabilities at present but will get better and become a core method.	Mostly rule based systems but some classification.	Improved techniques, apply to more languages than English.
Abd Rahman	Adequacy, challenges, and limitations of SGOPE data for detecting MH problems	Mental Health	Data Sources, Condition, location, Feature extraction methods, analysis methods	22 studies: stress 8, depression 7, suicide 3, MH disorders 4. Geographical: China 6, US, 4, Japan 1, Greece 1, unspecified 10. Source: twitter 8, Sina Weibo 5, Facebook 2, others 7. The keywords used to select data often not specified. SVM (13/22) most popular classification, LR & RF (5/22), NB 4/22)	Text analysis, multi method inc questionnaires, accessing respondents OSN accounts. Feature extraction TF-IDF, ngrams, BOW,	Multiple sources, other languages, inclusion of audio, video, photos. Better methods
Al-Garadi 2016 [25]	Adequacy / limitations of SM for pandemic surveillance	Infectious disease	Data source and volume, analysis method, study aims and outcomes. Features and classifier performance of supervised methods.	Can complement existing systems but still problems with representivity. Need better algorithms and computational linguistic methods.	Mostly supervised, classification. SVM. Most used ngrams as features.	Better algorithms/ computational linguistics
Allen 2016 [26]	Better understanding of how patients with chronic disease share knowledge in online spaces. Possibilities for improving self-management.	Chronic	Network themes and mechanisms	Helpful in encouraging patients to self-manage l/term conditions through sharing collective knowledge, gifting relationships, sociability and disinhibition. Need to understand why people do or do not post.	Qualitative: thematic, grounded theory, content & thematic, IPA, ethnography	Find out why people are reluctant to post and illuminate how these communities help people manage their condition in daily life.
Barros 2020 [27]	To assess research findings regarding the application of IBSs for public health surveillance (infodemiology or infoveillance). Sources, purposes, methods	Public Health	Paper type, year, disease, health topic, forecasting, surveillance, disease characterisation, first person health mention, diagnosis prediction,	Infectious disease the biggest area. We also identified limitations in representativeness and biased user age groups, as well as high susceptibility to media events by search queries, social media, and web encyclopaedias	Correlation analysis (59/162) regression models (46/162). Machine learning 27/162, statistical models 20/162. Manual analysis 18/162, topic analysis 12/162. Deep learning 10/162, linguistic analysis 10/162. Rule-based techniques (*n* = 7), epidemiology theory(*n* = 6), surveys (*n* = 3), and ranking techniques (n = 1) were used in less than 10 papers.	Updating keywords to reflect changing search behaviours and health trends. Susceptibility of SM content to media events. Creation of standard datasets to improve method development.
Calvo 2017 [20]	What NLP methods used on user generated data in mental health?	Mental health	Objectives of studies, data sources, features extracted	Triaging MH issues seems like a great use but need to find how to react to it in practice. Ethics/ privacy issues. Very interdisciplinary.	LIWC most widely used both for feature extraction and Sentiment analysis. Good methods often a combination of methods/ algorithms. Lots of different tools/ techniques available- could not determine whether any one was superior.	Need to do research into using NLP in different languages. Also think about how to make contact with people identified as being at risk from mental health that are identified during the process.
CastiiloSanchez 2020 [28]	What ML techniques used to predict suicide from SM data?	Mental Health	Methods, Tools, Techniques	Text classification main objective for 75%. 8/16 studies report explicit datamining techniques. 10/16 using SVM. Papers not reporting time spans of data collection, or number of participants.	LIWC, LDA, LSA for feature extraction, Sentiment analysis	Other languages. Use annotated corpus. Develop new tools. Do temporal studies.
Charles-Smith 2015 [29]	Can SM be used for disease surveillance? Or to test interventions to improve health outcomes?	Infectious disease	Correlation between social media data and national health statistics. Prediction times. Topic / theme identification. Influence on health behaviours.	Earlier prediction of outbreaks. Correlation with existing methods. Topic modelling good for broad topics, but not for lower frequency themes. Lots of gaps in knowledge. Need to look for ways to incorporate SM into PH surveillance.	Topic modelling (LDA). Query selection and thematic analysis to detect lower frequency topics.	Work on who uses what types of social media, so as to get representative data. SM platforms/ preferences change.
Cheerkoot-Jalim 2020 [30]	Identify the text mining approaches, tools used in biomedical text. Who benefits? Application areas? What are the challenges?	Any	Data Sources, Techniques, Tools and Potential Beneficiaries of research	Looked at who could benefit from SGOPE research	MetaMap, UMLS used - mainly on EHRs and biomed literature. NLP methods; NER and relationship extraction.	Big data paradigms, methods that can scale with the volume of text. Methods of standardising data across sources. Improving accuracy.
Convertino 2018 [31]	Summarise strategies, assess quality of information, potential for early detection from SM.	ADR	Sources, study population, drug Proto-ADE pairs, clinical features, extraction method.	Lots of potential to complement existing regulatory agencies. But utility, validity and implementation are all under-studied. Need standardised methods. Fast moving field. No causality assessment so far.	Keywords, dictionary most popular 37/38.	More work to improve methods. Use in conjunction with other signal detection methods.
Demner-Fushman 2016 [32]	Improvements in NLP on patient language, and new opportunities.	Any	SM as a source for quality assessment. Methods	Much more to be done both in clinical and SM NLP. Research moving from capturing trends to addressing individual health-related posts, thus showing potential to become a tool for precision medicine and a valuable addition to the standard healthcare quality evaluation tools.	Sentiment analysis. Rule based RegEx or supervised event extraction most used.. More work needed on semantic processing. Using sentences better than words,	Need more publicly available clinical datasets. Work on semantics. Work on porting pipelines across domains. Collaboration between NLP research and EHR suppliers.
Dobrossy 2020 [33]	Assess volume, participants and content of SM data about breast screening. Potential for patient education.	Breast Cancer	Platforms, volume of discourse, participant roles, discourse content, themes.	Looked at age, role of user types, and the content of the posts. Good source to understand beliefs, attitudes, and literacy of the target population.	NS	NS
Dol 2019 [34]	How health researchers are using SM data.	Any	Journals, study country, first author discipline, health topic covered, platforms, study purpose.	81/414 analysing content. Biggest use was recruitment. Generally seen as positive but concerns re ethics.	NS	Need methods to optimise usage and demonstrate potential.
Dreisbach 2019 [35]	Using NLP methods to extract symptoms from SM text	Symptoms	Study purpose, data source, symptom categorisation, evaluation, and performance metrics	Pain and fatigue most evaluated symptoms. Variety od sources. NLP primary methodology for 15/21 papers. Current focus on extraction of terms. Need to share lexicons to move forward.	21 papers: 14 NLP, 6 text mining, 1 NLP + TM. No breakdown of type of methods.	Future research should consider the needs of patients expressed through ePAT and its relevance to symptom science. Understanding the role that ePAT plays in health communication and real-time assessment of symptoms, is critical to a patient-centred health system.
Drewniak 2020 [36]	Does SGOPE research have quantifiable risks or benefits for patients, relatives, or HCPs?	Any	Purposes of the narrative: inform, engage, model behaviour, persuade, comfort	Generally positive benefits although potential risks from misinformation	NS	Future research is needed to define the optimal standards for quantitative approaches to narrative-based interventions.
Edo-Osagie 2020 [37]	Current uses of Twitter data in public health	Any	Conditions, data sources, analysis methods, geographical and time trends	Twitter a good data source for 6 aspects of public health: surveillance, event detection, pharmacovigilance, forecasting, disease tracking and geographical identification.	Numerous	Unsupervised methods. Do research into less studied areas
Falisi 2017 [38]	What role does SM play in the health of breast cancer survivors?	Breast cancer	Platforms, ethnicity of study population, analysis method, which aspects analysed, connection between SM content and health outcome.	Focus on psychosocial wellbeing. Mostly online support forums/ message boards. Few non-Caucasian. Content analyses of social media interactions prevalent, but few articles linked content to health outcomes	40/98 did content analysis. Some manual / some M/L. Pre 2011 = LIWC, post 2011 = LDA etc. 37 quant. 3 qual	Should consider connecting SM content to psychosocial, behavioural, and physical health outcomes. None of the content analysis articles attempted to do this.
Filannino 2018 [39]	What tasks and methods included in the shared tasks?	Any	Task description, data type, data source, dataset size, best performance, measure.	NER & classification the most used tasks. Clear trend to data-driven solutions. Need more and varied datasets to explore.	NER and classification most common tasks.	Bigger and more varied datasets to share
Fung 2016 [40]	What research questions and methods used on Ebola related social media?	Infectious disease	Study design, qual or quant, study aim, data collection method, time frame, keywords used, analysis method, main findings, and limitations.	12 papers: 8 from Twitter/ Weibo, 1 from Facebook, 3 from YouTube, and 1 from Instagram and Flickr. All studies were cross-sectional. 11/12 articles studied one or more of themes / topics of SM content, post meta-data and characteristics of the SM account. Twitter content analysis methods included text mining (n = 3) and manual coding (n = 1). Two studies involved mathematical modelling. YouTube /Instagram/Flickr studies used manual coding of videos and images. Published Ebola virus disease-related social media research focused on Twitter and YouTube. The utility of social media research to public health practitioners is warranted. No evaluation of the studies utility performed.	Mix of manual coding and frequency analysis using LIWC.	Need a new checklist to appraise quality of SM papers. Future research in the direction of analysing multiple cross-sectional social media datasets or conducting prospective cohort studies of social media users will provide useful data for analysis of temporal change of social media contents or social media users’ behaviours. Need to bridge research and practice.
Gianfredi 2018 [41]	Can SM be used for disease surveillance / predictions? Can they capture public reactions to epidemic outbreaks?	Infectious disease	Data source, disease, study period, geographical location, study purpose, type of analysis and main findings	Out of the 47 articles included, only 7 were focusing on neglected tropical diseases, while all the other covered communicable tropical/sub-tropical diseases, and the main determinant of this unbalanced coverage seems to be the media impact and resonance.	Qualitative, narrative analysis, content analysis, mathematical modelling, correlational analysis, geospatial.	Lots of gaps, possibly due to the media impact of the specific disease. Need further research into ways of integrating diverse data sources.
Giuntini 2020 [42]	Sentiment and emotion analysis for identifying depressive disorders. What types of SM data? Which networks? Which methods?	Mental Health	Platform, type of SM, emotion or feeling detection, other disorders inferred, methodology	Most used media is text, then emoticons. Twitter most employed platform. Supervised methods with off the shelf classifiers combined with lexicons such as LIWC.	Supervised (NB, DT, SVM etc) plus LIWC, NRC Word Emoticon, word-Net Affect lexicons	More multidisciplinary studies.
Gohil 2018 [15]	What sentiment analysis tools for Twitter / healthcare. Any health specific training, validation or justification	Any	Health area, sentiment towards, type of method, tool used, manual annotation sample size, sample size	Multiple methods mix of open source, commercial and bespoke tools. Very few tested for accuracy.	Sentiment analysis. Mix of tools.	This study suggests that there is a need for an accurate and tested tool for sentiment analysis of tweets trained using a health care setting–specific corpus of manually annotated tweets first.
Golder 2015 [43]	Prevalence, frequency and value of ADR comments from SM	ADR	Data source type, ADR type, search strategy used, post selection, study aim, ADR prevalence, comparison method	51 studies, discussion forums most used source type. ADR prevalence varied from 0.2 to 8%. General agreement that a higher frequency of adverse events was found in social media and that this was particularly true for ‘symptom’ related and ‘mild’ adverse events.	8/12 used Consumer Health Vocab dictionary. Few evaluation methods	A cost-effectiveness analysis of all pharmacovigilance systems, including social media is urgently required.
Gonzalez-Hernandez 2017 [44]	Show how NLP is developing in regard to capturing the patient perspective from unstructured text.	Any	Types of SM sites, analysis type, types of tasks.	Move from rule based to learning based systems. Work needed on noise reduction and normalisation/mapping. Shortage of annotated shared datasets. Shared tasks useful development tool.	Move from rule based to learning methods. Over 50% papers used lexical content analysis. In SM NLP: regex, LDA topic modelling. Supervised classification. Sentiment analysis	Normalisation of data, co-reference and temporal relation extraction. Need to create and release annotated datasets and targeted unlabelled data sets in distinct languages.
Gupta 2020 [45]	What methods, sources, are used for SM based health surveillance. Potential applications, and challenges.	Any	ML Methods, Data Sources, Diseases, Limitations of SM systems	Twitter most used source (64%). SVM most used method (33%) - better at binary classification.	SVM, Decision trees, random forest, NB, Logistic Regression	Noise reduction, Combining SM with other data, theme detection, develop better predictive models for epidemic prediction. Only 3 studies included ethical debate.
Hamad 2016 [46]	How is content analysis used in health-related SM studies?	ADR	Keywords and hashtags, sampling and data collection, analysis methods, validation, and presentation of results	Methods used were not purely quantitative or qualitative, and the mixed-methods design was not explicitly chosen for data collection and analysis. Proposes CCA analysis as straightforward method for Twitter analysis	Content analysis (quantitative and qualitative). Infoveillance. Combined content analysis (mix of mixed methods and content analysis)	NS
Ho 2016 [75]	Compares omics, social media and EHRs as sources of ADR knowledge	ADR	Study aims, Data & Tool, Method	Data driven approach essential to detect /predict ADRs. Omics data, EHRs and SM all new opportunities.	Datamining. NLP, NER, ontology building. Classification to exclude noise. Aims to reduce false positive rate. Yang = mix of topic + classification. Classification to link effect to drug. UMLS & MetaMap	NS
Injadat 2016 [47]	Techniques, areas, performance, comparison of techniques, strengths, and weaknesses of data mining methods.	Any	Domains, Techniques, Research objectives, Strengths, and weaknesses of techniques.	19 data mining techniques used to address 9 different research objectives in 6 different industrial and services domains. Most used methods: SVM, NB & DT. Most used in business and social network analysis. Medical/health use only 8%	Datamining. SVM, BN, DT	Research into how techniques are implemented. Need more statistical tests of results. But - many of the tests applied required a normal distribution which was not the case. Health researchers not good about writing about the methods used. Could learn a lot from CRM and HRM domains.
Karmegan 2020 [48]	Aims to analyse the possibility, effectiveness, and procedures of using SM data to understand the emotional and psychological impact of unforeseen disaster on the community.	Mental Health	Platform, methods	Twitter most used source. Sentiment analysis used for psychological surveillance. Could not conclude that any one method was superior.	Feature extraction using classification algorithms. Sentiment analysis	Combine text and image processing. Incorporate social network analysis with post content.
Kim 2017 [49]	How SM data can be used to understand communication and behavioural patterns of nonmedical or problematic use of prescription drugs	Substance misuse	User characteristics, communication characteristics, outcomes, methodological domain, ethical domain	See lots of potential, but more work needed.	Mixture: manual, qualitative, supervised / non supervised ML to identify themes, patterns, sentiment.	Lots more - sees their review as a base to build on. Identified a lack of theoretical framework for substance misuse monitoring. Consequences of SM engagement understudied.
Lafferty 2015 [50]	How is SM being used in psychiatry? Tools, benefits, and challenges.	Mental health	SM as data, methodological considerations, ethical considerations, SM for recruitment	Observational, real time patient experiences. Can help with development of practice, policy, and provision. Opportunities for co-creation of research, patient centric care.	Grounded theory, Social network analysis	Ethical issues. Analyse SM data through different socio-cultural lenses to build theoretical frameworks.
Lardon 2015 [51]	Can SM be a new source of knowledge for pharmacovigilance?	ADR	Language, data source, data volume, methods, lexicon,	Identification theme all 11 papers used manual methods. Identified heterogeneity of methods, but also gaps. Included studies failed to assess the completeness, quality, or reliability of the data.	RQ1: All manual /mixed, RQ2: Web scraping, pre-processing, various rule-based methods.	Additional studies are required to precisely determine the role of social media in the pharmacovigilance system. Need methods to assess data quality.
Lau 2019 [52]	2018 SOTA of opportunities, challenges, and implication of AI in health informatics	Any	NS	Few 2018 papers reported Artificial Intelligence (AI) research for patients and consumers. No studies that elicited patient and consumer input on AI. Most common use is secondary analysis of social media data (e.g., online discussion forums). The 3 best papers shared a common methodology of using data-driven algorithms (such as text mining, topic modelling, Latent Dirichlet allocation modelling), combined with insight-led approaches (e.g., visualisation, qualitative analysis, and manual review), to uncover patient and consumer experiences of health and illness in online communities. There is a lack of direction and evidence on how AI could actually benefit patients and consumers.	Best papers shared a common methodology of using data-driven algorithms (such as text mining, topic modelling, Latent Dirichlet allocation modelling), combined with insight-led approaches (e.g., visualisation, qualitative analysis, and manual review), to uncover patient and consumer experiences of health and illness in online communities	See what patients want from AI in health. More patient involvement to ensure that research is asking the right questions.
Lopez-Castroman 2019 [53]	Detecting suicide ideation from SM	Mental health	NS	Early days, but SM has important role in suicide prevention. Lots more work needed.	Various: Sentiment analysis, topic modelling, data mining	Add demographic data to text to improve results.
Mavragani 2020 [54]	Current state of SM based infodemiology. Validity of methods and research gaps.	Any	Timeline & journals. Data sources, Health topics, Advantages & Disadvantages of SM data	JMIR most used journal. Increasing interest since 2018. Twitter most used platform. Most researched subjects were conditions/diseases, epidemics, healthcare, drugs, smoking/alcohol.	NS	Combine SM data with traditional sources for more complete assessment.
Neveol 2017 [55]	Best clinical NLP papers of 2016	Any	Applications of NLP, Directions of progress	Developing applications rather than methods. Starting on the more complex tasks e.g., semantics, coreference resolution, and discourse analysis.	Classification of useful sentences, Information extraction, abbreviation disambiguation, coreference resolution, grounding of gradable adjectives	NS
Neveol 2018 [21]	Summarize recent research / best papers for clinical NLP in 2017	Any	NLP of SM data, NLP of HCP text, methods	2017 trends - revisiting old problems such as SM classification and negation with deep learning & neural nets. Production of annotated corpora. Continuing applications rather than methods. Beginning of deep learning. Start of language variants.	Negation detection, corpus annotation, deep learning.	Work in other languages. Increase generalisability.
Patel 2015 [56]	Categorise & summarise existing papers about chronic disease outcomes from SM. Suggest framework for future research.	Chronic	Platform, Taxonomy category, disease, study aim, study design, sample size & description, Method summary, SM effect	85% either Facebook or blogs. 40% for support (social, emotional, or experiential).	Quantitative, Thematic qualitative, Content analysis.	Understand how disease, patient factors and tech can interact to improve outcomes. Reduce potential for bias. Target studies to specific diseases might be the best way to improve clinical care.
Pourebrahim 2020 [57]	Datamining methods for ADR detection from SM	ADR	Analysis and evaluation metrics	SM good for early identification of ADRs. Three main stages; Pre-processing, feature extraction and classification	Supervised, regression, unsupervised	NS
Qiao 2020 [58]	Overview of SM studies relating to mental disorder detection.	Mental Health	Platforms, collection methods, feature extraction, algorithms, evaluation metrics	Facebook, Twitter, Reddit, Tumblr, Instagram. Most used supervised methods, especially SVM	SVM, Decision trees, random forest, NB, Logistic Regression	Develop systems with lower computational cost to increase speed. Multi-language systems.
Ru & Yao 2019 [7]	SGOPE data - methods/analysis opportunities and challenges	Any	Data type, volume, pre-processing method, analysis method, health outcomes	Variety of methods. Outcomes included side effects / effectiveness / adherence / hrqol	NER, mapping, identify concepts, text mining (Ngram, LDA, topic modelling), content analysis, hypothesis testing, supervised, unsupervised	Suggested further research on treatment effectiveness, adverse drug events, perceived value of treatment, and health-related quality of life. The challenge lies in the further improvement and customization of text mining methods. Only 6 discussed ethics.
Santos 2019 [59]	Numbers of papers / journals, countries / databases, methods/tools, which public health issues looked at	Public health	Year, Journal, Study purpose, health area, techniques, software/ programming language, study country	Results showed a slight increase in the number of papers published in 2014 and a significative increase since 2017, focusing mostly on infectious, parasitic, and communicable diseases, chronic diseases, and risk factors for chronic diseases. JMIR and PLoS ONE published the highest number of papers. Support Vector Machines (SVM) were the most common technique, while R and WEKA were the most common programming language and software application, respectively. The U.S. was the most common country where the studies were conducted. In addition, Twitter was the most frequently used source of data by researchers.	SVM, Decision trees, random forest, NB, most used techniques. R, WEKA, and Python most used languages/ apps.	In depth analysis of variations in techniques (deep learning / ensemble etc)
Sarker 2019 [60]	Look at existing methods of SM based medication abuse or misuse, propose new data centric pipeline.	Substance misuse	Data source, dataset size, medication studied, study objectives, methods, and findings.	39 studies, 80% published since 2015. Twitter most used source. Earlier studies manual qualitative, but growing trend towards NLP methods.	Supervised, unsupervised	Develop shared annotation guidelines and annotated datasets. Will help the direct project and enable comparison across methods. Show agreement for manual annotation. Reduce noise in data.
Sharma 2016 [61]	Identify and highlight research issues and methods used in studying Complementary and Alternative Medicine (CAM) information needs, access, and exchange over the Internet.	CAM	NS	Significant interest in developing methodologies for identifying CAM treatments, including the analysis of search query data and social media platform discussions	Qualitative, thematic, content analysis, keyword searches, regex, Consumer health vocabulary	Little work done on using SGOPE to understand CAM user’s perspectives / prevalence of CAM use. Lots more work required.
Sharma 2020 [62]	Can sentiment analysis be conducted on social media platforms to understand public sentiment held towards pharmacotherapy?	Any	Author, Year, Journal, data source, conditions, pharmacotherapy, SA method used, potential clinical use.	Lack of consistent approach. Opinion on particular medication (7/10) and ADRs (3/10) Lexicon based more used than ML for sentiment. (Lexicon 6, ML 1). Combining SA with other ADR methods improved results. Lots of untapped potential.	Lexicon, ML. Combining	No gold standard methods yet. Early stage of development. Accuracy rarely assessed.
Sinnenberg 2017 [63]	How and why health researchers using Twitter?	Health research	Ways Twitter data used by researchers, ways that Twitter platform used in health research, Publication date, research topic, ethics, and funding	The primary approaches for using Twitter in health research that constitute a new taxonomy were content analysis (56%; *n* = 77), surveillance (26%; *n* = 36), engagement (14%; *n* = 19), recruitment (7%; *n* = 9), intervention (7%; n = 9), and network analysis (4%; *n* = 5).	Content analysis, network analysis	Future work should develop standardized reporting guidelines for health researchers who use Twitter and policies that address privacy and ethical concerns in social media research. New opportunities to characterise users from metadata such as demographics.
Skaik 2020 [64]	Recent trends and tools for using social media posts to predict mental disorders using ML and NLP methods. Identifying research gaps.	Mental Health	Collection methods, applications, best practices, and gaps	25 papers looking at population level mental health classification techniques. 15/25 depressive disorders, 10/25 suicide-ideation. Twitter most used data source, SVM most used model. Heterogeneity of methods and feature selection.	Models: SVM, Ensemble, LR, RF, DT, LSTM. Features: WEKA, LDA, TF-IDF, Sentiment, Lexical, Syntactic, Demographics, Word embedding, Topic modelling	Improve identification of risk factors.
Staccini 2017 [65]	Uses and challenges for secondary use of health data	Any	Data donation, uses of SGOPE data	Secondary use of patient data (apart from personal health care record data) can be expressed according to many ways. Requirements to allow this secondary use should be harmonized between countries, and social media platforms can be efficiently used to explore and create knowledge on patient experience with health problems or activities. Machine learning algorithms can explore those massive amounts of data to support health care professionals, and institutions provide more accurate knowledge about use and usage, behaviour, sentiment, or satisfaction about health care delivery.	NS	Very early days, lots to work on. Socio-ethical concerns, increased adoption in health care. Need to check AI /SM is asking the right questions. Need a formal framework for consent and secondary use of data. Far from massive adoption in health practice.
Su 2020 [66]	Deep learning in Mental Health	Mental Health	Methods, Tools, Techniques	A growing number of studies using DL models for studying mental health outcomes. Particularly, multiple studies have developed disease risk prediction models using both clinical and non-clinical data and have achieved promising initial results. Lots of potential but lots of challenges	CNN, RNN, Autoencoders	Reduce bias, improve methods
Tricco 2018 [67]	Using SGOPE for ADR detection. Types / characteristics of platforms? How valid or reliable are the conversations?	ADR	Data sources, document characteristics, health conditions, methods, types of listening system, outcome results	46/70 documents (66%) described an automated or semi-automated information extraction system to detect health product AEs from social media conversations (in the developmental phase). Seven pre-existing information extraction systems to mine social media data were identified in eight documents. 19/70 documents compared SM reported AEs with validated data: consistent AE discovery in 17/19. No evaluation of methods or reliability.	Supervised 15/70, Rule based 6/70, unsupervised 4/70, deep learning 1/70, other ML 5/70, Manual or NA 32/70. Dictionary/ lexicon based most used.	Further research is required to strengthen and standardize the approaches as well as to ensure that the findings are valid, for the purpose of pharmacovigilance. Studies required to look at uses / utility over a longer time period. Need standardised methods. Fast moving field.
Vilar 2018 [68]	To review datamining as a method of detecting drug-drug interactions from pharmacovigilance sources, scientific literature. Challenges and limitations compared.	ADR	Data source, methods	SGOPE offers new possibilities for identifying DDIs. Current emphasis has been on ADRs not DDIs.	Dictionary matching, association mining, supervised LR.	More studies are necessary to really prove and understand the potential of social media resources and their role in pharmacovigilance.
Wilson 2015 [69]	Understanding how blogs could be used for qualitative health research	Any	Geographical location, study aims, now data used in health research.	Used for data collection and recruitment. Good for accessing out of reach populations. Potential for significant improvement of health equity. Sees blogs as ‘central part of global transformation’. Need to develop knowledge and skills to take advantage of this new resource.	Purely qualitative	Look for innovative methods to develop qualitative research.
Wong 2018 [70]	To review methods of identifying adverse events from free text	ADR	Definition of NLP tasks, evaluation metrics, challenges in applying NLP to medication safety, data source, methods	Time saving/ real time. Limited by lack of data sharing inhibiting large-scale monitoring across populations. SM good for groups such as children, pregnant women, often not included in trials. Data is Pt reported outcomes, values / preferences - more patient focused.	Supervised, CRF classifier, unsupervised k-means clustering. Linguistic based, standardising text with UMLS. Statistical based.	Integrate data sources from different domains to improve ADR detection. Ethical issues. Increased volume of open-source data.
Wongkoblap 2017 [71]	Scope & limitations of new predictive method using SM. Ethical concerns.	Mental health	Key characteristics, data collection techniques, data pre-processing, feature extraction, feature selection, model construction, and model verification.	Methods work across languages. Despite an increasing number of studies investigating mental health issues using social network data, some common problems persist. Assembling large, high-quality datasets of social media users with mental disorder is problematic, not only due to biases associated with the collection methods, but also with regard to managing consent and selecting appropriate analytics techniques.	Most common method was text analysis with LIWC. Sentiment analysis. Supervised / predictive models. Only 1/58 used deep learning,	Move towards open science standards - share datasets / workflow /code. Ethical aspects of using SM data not clearly defined. Lack of models for detecting stress or anxiety disorders. Combining SM content with confirmed patients rather than self-reported ones. Network analysis to investigate prevalence.
Yin 2019 [16]	To systematically review the effectiveness of applying machine learning (ML) methodologies to UGC for personal health investigations.	Any	Methods, Objectives, Data Source, Health issue, Language, Dataset size	103 eligible studies, summarized with respect to 5 research categories, 3 data collection strategies, 3 gold standard dataset creation methods, and 4 types of features applied in ML models. Popular off-the-shelf ML models were logistic regression (*n* = 22), support vector machines (*n* = 18), naive Bayes (*n* = 17), ensemble learning (*n* = 12), and deep learning (*n* = 11). The most investigated problems were mental health (*n* = 39) and cancer (*n* = 15). Common topics were treatment experience, sentiments, and emotions, coping strategies, and social support. Clinical credibility an issue. Application in practice - who should monitor UGC. Conflicting advice from peers / HCPs a potentially interesting avenue. SGOPE can learn health information not in EHRs. Processing and ethical challenges unresolved.	Logistic regression (22), SVM (18), Naïve Bayes (17), ensemble learning (12), deep learning (11)	Ethical aspects of analysing personally contributed data, bias induced when building study cohorts and dealing with natural language, interpretation of modelling results, and reliability of the findings.
Zhang 2018 [72]	Consideration of Twitter as a data source for health researchers.	Any	Research design, collection techniques, analytic methods, tools, author’s opinion on Twitter as a health research method.	17 papers: Quantitative (n = 2), qualitative (n = 7), and mixed methods (*n* = 8). Health topics and research questions included pain, migraines, and cancer, social discourse of conditions like perceptions of portrayal of seizures, and cyberspace compared to real-world phenomena. Twitter currently used to search and mine research data. Utilizing Twitter as a recruitment and data collection tool in health research remains largely unexplored. Data collection predominantly passive and covert data collection. Challenges include verification, ethics - overt or covert collection.	Qualitative, quantitative, mixed methods	Creates new questions about data collection, verification, ethics for researchers.
Zhang 2020 [73]	Role of SM, themes and methods used in SM based public health research.	Any	Publication trends, themes, role of SM, research methods	Growing number of publications and journals including studies.	Still mostly qual or quant, with little use of computational methods.	Need to develop the methodological potential.
Zunic 2020 [74]	Data sources, roles, motivations, and demographics of posters. Topic areas. Practical applications, methods and current performance levels of sentiment analysis.	Any	Data sources, role of post author, demographic features recorded, health area, ML algorithms used for SA, classification performance, lexical resources	86 studies. Majority of data from social networking/ Web-based retailing platforms. Primary purpose of online conversations is information exchange/social support. Communities tend to form around health conditions with high severity / chronicity rates. Topics include medications, vaccination, surgery, orthodontic services, individual physicians, and health care services in general. 5 poster roles identified: sufferer, addict, patient, carer, and suicide victim. Only 4 reported demographic characteristics. Many methods used for SA. Mainly supervised. Only 1 study used deep learning. Performance less than achieved by general sentiment analysis methods. F-score, below 60% on average. Few domain-specific corpora and lexica are shared publicly for research purposes. Unclear if performance issues are because of the intrinsic differences between the domains and their respective sublanguages, the size of training datasets, the lack of domain-specific sentiment lexica, or the choice of algorithms.	Sentiment analysis. Mix of tools. A wide range of methods were used to perform SA. Most common choices included support vector machines, naïve Bayesian learning, decision trees, logistic regression, and adaptive boosting. Only 1 study used deep learning.	Improved methods. Performance less than achieved by general sentiment analysis methods. Lack of domain specific datasets / lexicons. Need to create and share large, anonymised domain specific datasets. More inclusion of demographic data.

In terms of the specific health topic of interest, 22/58 papers included any health condition [7, 15, 16, 21, 30, 32, 34, 36, 37, 39, 44, 45, 47, 52, 54, 55, 62, 65, 69, 72–74]. Twelve focused on mental health conditions [20, 23, 24, 28, 42, 48, 50, 53, 58, 64, 66, 71], 9 on adverse drug reactions (ADRs) [31, 43, 46, 51, 57, 67, 68, 70, 75], 4 on infectious diseases [25, 29, 40, 41], two each on chronic disease [26, 56], substance misuse [49, 60], public health [27, 59], breast cancer [33, 38] and with one each for symptom identification [35], use of complementary and alternative medicine (CAM) therapies [61] and the reasons for existing use by health researchers [63].

As a retrospective surveillance tool SGOPE has been used to capture public reaction to health events in terms of emotions [20, 42], fears, knowledge [26], attitudes and behaviours [27, 41]. Karmegam [48] looked specifically at studies evaluating the potential of SM data to understand the emotional and psychological impact of unforeseen natural disasters in a community. Several reviews focus on using SGOPE data to monitor behaviours, communication patterns and spread of health related concepts, particularly relating to infectious diseases [25, 27, 29, 40, 41]. Both the speed and accuracy of tracking are seen to improve on existing surveillance and signal detection systems, although most conclude that SM surveillance should currently be complementary to existing systems rather than replace them [25, 29, 31, 67]. Analysis of SGOPE data has been used to understand the various network mechanisms of information spread, the topics that are discussed, and to identify trends or patterns within the conversations [23, 24, 26, 33, 34, 41, 43, 48–50, 54, 61, 63, 67, 73].

A study on chronic disease collated qualitative studies exploring how people shared knowledge within the communities to show how the distinct characteristics of online spaces helped patients self-manage their long term conditions in ways that are difficult to replicate off line, and how these spaces were filling an unmet need for information and or emotional support [26, 36].

One of the most frequent use specific use cases was as a new source for identifying adverse drug events or reactions [16, 31, 43, 51, 57, 67, 68, 70, 75]. Identified advantages of SGOPE data over existing sources include earlier identification of ADRs [31, 43, 68, 70], the reduction of associated economic costs and potential fatality numbers [57] and the highlighting of ‘mild’ adverse events that may not be seen as serious enough to report through existing routes. Golder [43] found that the prevalence of adverse event reporting on SM ranged from between 0.2 to 8% of the posts, with ‘mild’ events being over represented, while ‘serious’ ones were under represented as compared to other ADE discovery methods. Comparisons between the data sources show that SGOPE data is generally in concordance with other regulatory sources for most adverse events [43, 67], but that at this early stage of method development that it should be used in conjunction with other existing methods [68, 70]. Combining SGOPE with EHRs and omics data is seen as an essential method of detecting and predicting ADRs [75]. The additional context from the patient experience narrative adds to existing post-marketing surveillance of interventions [16, 67].

Two reviews looked at the misuse of prescription medicines [49, 60]. Kim [49] used findings from existing Twitter analysis to create a typology of SM big data analysis on the topic based on the four conceptual dimensions of poster characteristics, communication characteristics, predictors and mechanism for the discussion of problematic use, and the psychological or behavioural consequences of discussing it on social media.

Other use areas included assessing the opportunities, benefits, challenges and limitations that using SGOPE data might offer healthcare providers and researchers [23, 25, 36, 37, 42, 45, 52, 57, 62, 64, 71, 73]. Benefits identified included providing a new channel for hearing patient perspectives of their health experiences [23, 33, 42], faster data collection and reduced costs [25, 33], and improved support for self-management of health conditions [26]. Zhang [73] categorised SM based papers by their role in public health, with the most frequent use case being as an interactive intervention tool aimed at modifying risky health factors. Classifying studies into five categories encompassing, education, disease modification, diagnosis, support and management, Patel [56] evaluated the impact of social media use on outcomes across a range of chronic conditions, concluding that few studies suggested any harm from its use and that as a data source it had tremendous potential to improve patient care. Drewniak [36] looked at the risks and benefits of using patient narratives for patients, relatives and HCPs, finding that they were a promising way of improving patient understanding of their health conditions capable of impacting behaviours and outcomes.

Ru [7] concluded that with improvements in analysis methods, findings from SGOPE would be able to generate new research questions around effectiveness, ADRs and health related quality of life.

Vilar [68] evaluated SGOPE as a method of identifying drug-drug interactions (DDI). They conclude that existing DDI resources such as DrugBank, Micromedex and DDI Corpus, although good as knowledge or evaluation bases show little consistency, and that SGOPE had the potential to be instrumental in creating knowledge sets and identifying unknown DDIs.

Calvo looked at the ways and levels that NLP could be used within mental health, including triaging people at risk and diagnosis of specific conditions. At a post level, emotions and risks can be identified, temporal changes can be tracked at the author level, and general trends in sentiment and attitudes established at a population level [20].

One review identified how language markers, such as higher use of pronouns, can be indicators of altered mental state or suicide ideation [21]. Using predefined semantic vocabularies allowed the identification of posts indicating both medium and severe mental illness [16].

Yin [16] looked specifically at SGOPE data as a route to understanding poster experience of health issues, concluding that it gave insights into health factors that often were not recorded in EHR systems. They summarised 103 papers into 5 research categories; those characterising health issues and patients, prediction of events such as suicide, the correlation between SM posts and existing data collection methods, those characterising drug usage/ adverse events/ misuse and detecting sentiment about major health events such as post-partum depression and how this impacted on posting behaviours. Recognising that symptom discussion is a large component of SGOPE data, one review focused on papers for symptom extraction [35]. Understanding how symptoms cluster is a recognised knowledge gap [76]. While pain and fatigue were the most common symptoms that were identified, many of the included papers in this review identified symptoms from 10 of the 12 symptom categories the review authors had previously defined, concluding that SGOPE data could help with faster diagnosis and understanding issues such as the recent opioid crisis and pain management.

### RQ3: analysis methods identified by the reviews

Analysis methods have varied widely as new tools and techniques have been developed, and the reviews reflect this [51]. Eleven of the reviews focused on the methods utilised to analyse this type of online data, while 34 looked at both uses and methods (Table 4). A study covering 2003 to 2017 highlighted the absence of specific trends in either approach, evaluation or performance [35].

This review found that many papers, even recent ones are still using traditional qualitative [26, 56, 69, 73] or mixed methods [41, 46, 50, 72] of analysis on small quantities of data. Of the 42 papers in the Patel review [56], only 3 analysed over 1000 posts, with 26/42 analysing less than 100 texts. Other reviews included papers using a mix of manual and machine learning methods [29, 38, 40, 49, 51, 61]. Abbe [23] argued that while the debates about qualitative and quantitative analysis continue, the exploratory yet highly automated approach of natural language processing (NLP) can bridge the gap, offering the best of both worlds.

Among the analysis methods used, sentiment analysis was the most commonly utilised [15, 16, 20, 32, 37, 42–44, 48, 49, 53, 61, 62, 64, 71, 74]. Our review found that much early sentiment analysis was often performed on small volumes of text, using qualitative or content analysis methods [46, 60]. Developed originally as a marketing tool for business to understand consumer opinion towards their product [15], sentiment analysis has frequently been used to identify emotions that can signify a posters thinking and mood when trying to identify potential suicide risk [15, 20, 28, 53], to track ADRs and to interpret patient reviews of health care services [74]. Simple automated content analysis has used lexicon based keyword techniques such as the LIWC (Linguistic Inquiry and Word Count) text analysis tool to count the frequency of keywords within the text [38] or compute the percentage of positive or negative emotional terms in a text [20, 29].

Machine learning methods have a myriad of different algorithms and techniques of varying levels of complexity in various stages of development. At a basic level they can be divided into either supervised (classification) or unsupervised (clustering) methods. Classification methods were often rule based, looking for a predefined words or patterns of text and the accuracy of the model is heavily dependent on the initial parameters in the choice of words or expressions [32]. The majority of the machine learning studies to date have used supervised methods. Common classification algorithms include Support Vector Machines (SVM), Naive Bayes (NB), Decision Trees (DT) and Random Forest (RF). All these and others are frequently mentioned in the method discussions although SVM is the most popular [25, 47, 59, 71, 74]. Gupta [45] noted that SVM was the most promising method for binary classification tasks. Unsupervised techniques using topic modelling which do not require large amounts of labelled data are beginning to become more prevalent, especially for identifying themes and topics within large quantities of text [29, 38] but were less frequently utilised [37]. A comparison of all datamining techniques found that they all had various strengths and weaknesses and that research objective and data should guide the choice of method [47].

The methods of both SGOPE and clinical NLP (looking at the unstructured text in EHRs etc.) have similar issues and purposes, but although automatic methods of processing are developing, the unstructured nature, noise, domain specific content, problems with language usage, understanding semantics and the complexities of informal speech mean that there is still a lot of work to be done in developing methods to maximise its usage [44, 45, 66]. Sarker [60] categorised the methods currently used to identify and monitor such use, concluding that there was still a lack of datacentric pipelines, and proposing a new method based on shared annotation guidelines and labelled datasets. One solution to the issues of ‘noise’ and irrelevant text within SGOPE is to use a combination of methods to refine the content by using a binary classification method to exclude any irrelevant content and then topic modelling to identify themes from the useful content [40, 44, 60]. Health domain language can be quite specific to the domain, even at a lay person level. A variety of approaches are being explored to deal with the inconsistencies of ‘patient language’ such as spelling correction, and attempts to map lay language to medical ontologies [32].

Shared tasks and datasets have been identified as a means of improving method development. Table 6 lists details of some of the SGOPE shared dataset challenges held to date that were identified in the reviews [39, 44]. Often used in computer science these approaches are seen as the most comprehensive way to evaluate methods and techniques. All groups taking part in each challenge share the same training dataset, and then develop algorithms or pipeline processes with the accuracy of each method calculated with the test data [20]. This allows an open assessment of the various approaches. Results from these suggest that the best results are often achieved by combining methods in a pipeline process, but that there is no one single combination that is seen as being the most effective [20]. Tasks such as entity recognition, especially using dictionaries, are much easier than the more complex problems of correctly identifying relationships between the entities. Reports from some of the shared task challenges show that within clinical NLP, named entity recognition (NER) can achieve an accuracy of over 70%, but relation extraction methods are much less successful with performances below 50% [39]. These figures are likely to be lower in SGOPE data where the variations in language, grammar and sentence construction are much wider.

**Table 6:** Shared dataset NLP challenges since 2015

Event	Data Source	Task	No tweets / posts	Best result	Methods used	Data availability
2015 CLPsych	Twitter	Binary classification of users based on depression / PTSD. 1. Depression vs control 2. PTSD vs control 3. Depression vs PTSD	7.857 million	Average precision 80%	SVM /TD-IDF weighting	With IRB approval & privacy agreement
2016 CLPsych	ReachOut forum	Classify triage level (1–4) for professional support	65,024	F1–42%	Variety of classifiers	With IRB approval & privacy agreement
2017 CLPsych	ReachOut forum	Classify triage level (1–4) for professional support	157.963	F1–46.7%	Variety of classifiers	With IRB approval & privacy agreement
2016 SMM	Twitter	1. Classify ADRs. 2. Map to UMLS (NER) 3. Concept normalisation	10,822	F1–42% F1–61% No result	Random forest (ngram) CRF	Yes
2017 SMM	Twitter	1. Classify ADRs. 2. Classify drug intake. 3. Concept normalisation	15,717 training 9961 testing	1. F1–43.5% 2. F1–69.3% 3. Acc −88.5%	SVM CNN LR/DeepLearn	Yes
2017 NTCIR-13	Twitter	Label disease / symptoms	2560 (English, Japanese & Chinese)	Exact match accuracy of 88%	Hierarchical attention networks (HAN) plus CNNs	Training data only

Adapted from [39, 44]

The latest developments in NLP move from rule-based systems to deep learning [16, 21, 37, 44, 74]. These methods aim to improve on the semantic level of understanding, by using language models such as word embeddings and distributional semantics [70]. Based on artificial neural networks, deep learning uses ‘hidden layers’ to extract more detail from the raw input. Within healthcare it is deep learning techniques that are behind the recent advances in automated image processing. As yet they seem to be rarely used within text based healthcare analysis, with only 1/86 papers using sentiment analysis methods using deep learning and 4/86 using word embeddings [74] although one review commented on how researchers were starting to use these methods on existing classification and negation identifications problems [21]. Only one review focused on deep learning methods, but these were mostly applied on EHR and biomed literature data with only a few examples of SGOPE data usage [66].

### RQ4: gaps and future research needed

All the reviews acknowledge that method development is still at an early stage and that much more work is needed before the full potential of SGOPE can be utilised. Particular challenges include algorithm design [25, 59], method refinement [51, 72] integrating diverse data sources [41, 70], pre-processing, coreference and temporal relation extraction [44, 51], spelling correction, normalising poster language [51], and reducing bias [16, 56, 66, 71]. Studies to date have considerable heterogeneity in methods and outcomes, further work is also needed to define the optimal standards for these [15, 28, 30, 31, 40, 51, 63].

Sentiment analysis performance on health related text was found to be lower than that of other domains [74] but that may be because most of the commonly used sentiment lexicons have been developed from publicly available film or restaurant reviews, but these do not work as well on health topics [74]. There are calls for the development of annotation guidelines [60] and sentiment analysis tools trained on health care specific corpora [15]. One of the problems with current standard sentiment lexicons is that they are too general for health topics. Attempts to map them to the Unified Medical Language System (UMLS) found that less than 1% of its content is covered by common existing lexicons [74].

At this early stage of method development there are a variety of tools and algorithms available to analyse unstructured text, but a lack of studies that compare their efficiency or accuracy [15] and therefore a lack of consensus as to which are the most useful [15, 43, 74]. Several reviews suggest greater sharing of datasets [32, 39, 44, 71, 74] and the wider development of shared tasks, where different groups can work towards solving a particular task on the same dataset [27, 32, 39, 44, 60].

The frequent lack of clear explanation of the methods used in studies [28, 47] and the poor reporting of datasets used [28] means that it is hard to assess the accuracy of many results and may lead to selective outcome reporting or publication bias [43].

Further work is needed in terms of evaluating the findings from SGOPE data, both against existing signal detection methods [31], and to psychosocial, behavioural and physical outcomes [38]. Comparisons of SGOPE data to that in clinical text such as EHRs or biomedical literature identified the potential value of SGOPE but highlighted the particular issues of noisy, irrelevant content, language inconsistencies and ambiguity [23, 32, 44, 55], but made no comment on how the accuracy of SGOPE data analysis compares with these methods [7].

Other areas for future research identified include the need to adapt the methods to languages other than English [20, 21, 23, 24, 28, 44, 58], cost-effectiveness studies [43], better understanding of how SGOPE can help posters self-manage [26], maximising the representativeness of the data [29, 35], facilitating evaluation [61, 67], integrating SM text with audio and video sources [24, 45, 48], and a better understanding of how SGOPE could integrate with existing systems [51]. Three reviews commented on the lack of demographic analysis, despite geotags being easily accessible from Twitter data [53, 63, 67, 74]. Each individual tweet has potentially 38 data features including detailed metadata such as geotags, but these seem to be unexplored at present [63]. Methods that included temporal analysis could help identify event sequences and causal inferences [28]. Only one review focused on health outcomes [7]. A lack of linking SGOPE interactions and analysis with health outcomes has also been identified [38].

There is a lack of both theoretical [49] and methodological data centric frameworks [60] for SGOPE usage, hindered by the discipline boundaries where researchers in one area often do not know of relevant literature in another. This is compounded by differences in language, terminology and methods that exist [20]. Giuntini suggests that a multi-disciplinary approach could help develop better algorithms [42]. The need for interdisciplinary collaboration between NLP and health researchers in order to maximise the opportunities available is highlighted [20, 32]. A gap between academic NLP research and the commercial NLP systems as beginning to be used on electronic health records (EHRs) has been identified, in that academic work tends to be more advanced [44]. One review looking at the development of methods identified a number of approaches that were in development for analysing SM text but concluded that many NLP developments are not getting as far as being used in applications – ‘they are often explored, published and then shelved’ [32].

Concerns around the ethics of using social media data posted in public spaces are ongoing and several reviews mentioned the need to be aware of ethical issues [16, 20, 28, 34, 49, 50, 63, 65]. The absence of any form of discussion around the ethical implications of this form of data use was highlighted in 23/26 surveillance studies [45] and 13/16 studies on suicide ideation [28]. The need for guidelines and harmonisation of regulation around secondary use of SM data and data donation was identified [65, 72] together with a call to analyse data through different socio-cultural lenses [50].

As issues around privacy and consent begin to be resolved, further questions emerge about the how findings should be incorporated into health care practice [35, 51, 52, 65]. Regarding its use as a method of public health surveillance there is a lack of guidance as to how health organisations should accept or react to data from SM discussions [29]. In the area of mental health questions remain as to if and how any posters deemed to be ‘at risk’ should or could be contacted [20].

This type of data source has traditionally been seen as lacking credibility, although recently several of the major science journals have begun to publish articles supporting its use within health research [34]. One identified limitation is the potential for the content to be influenced by media events or coverage [27, 29]. To increase its acceptability efforts need to be made to bridge research and practice by demonstrating how the research can translate into practice [16, 40]. Aligning SGOPE data with clinical EHR data could help to both bridge a credibility gap and help both posters and clinicians reach a better understanding of how health issues impact on individual’s lives [16].

## Discussion

In total, 58 review papers were included that answered the research question of how and why SGOPE data is being used in health research in terms of the sub questions. Of these, 13/58 looked primarily at the purposes, 11/59 primarily at the methods, while 34/58 addressed both the purpose and the methods. Despite the heterogeneity of studies included, the early stage of methodology development and the many challenges still to be overcome, there was universal agreement between them of the potential of SGOPE data to improve health and deliver patient centred care. Twitter is the most widely used data source, and the majority of studies to date have used either qualitative, quantitative or supervised machine learning methods. The growing significance of this type of data source is reflected in the volume of published literature especially since 2017.

### RQ1 which SGOPE sites / platforms are being used as data sources?

The high prevalence of Twitter as a data source probably reflects the easy accessibility to large volumes of data that has been accessible through their API, rather than its suitability for health research. Facebook was another common source, but recent privacy issues have resulted in far fewer messages being publicly accessible in recent years [15]. Access to some of the potentially more useful online forums and communities is being restricted, due to a combination of privacy concerns and commercial interests, as the economic value of health data is increasingly recognised [77].

Restricting individual studies to a single data source may be simpler for method development, but it does decrease the overall validity of the studies due to the elements of emotional contagion or other bias [78, 79] that may be present on a single data source, especially if it is a relatively small community. Even a massive source such as Twitter is still quite limited in the demographics of its posters which has implications for the generalisability of findings based on it [50].

### RQ2 what purposes is SGOPE data used for?

Although SGOPE data adds a new dimension to healthcare research [11], the topics that have been researched to date reflect both the early stages of methodology and the type of posts that are most available. The most active communities are known to be those with long term conditions or rare diseases [74]. Simple key word searches are easy to implement and can be very effective when searching through large volumes of text for mentions of selected drugs or conditions, but as methods develop the range of use cases will widen. Much of the use to date has been retrospective or evaluative, but as methods improve higher degrees of semantic meaning can be accurately extracted, and its role as a predictive or triage tool may become more widespread. One of the potential problems of studies based solely or mainly on one data source such as Twitter is that the content posted there tends to be heavily biased by media coverage of events, so that potential use cases for research is driven by the availability of the data [27, 41].

### RQ3: which analysis methods are being used in the studies?

The level of detail reported varied widely between reviews, with some of the reviews that looked at both uses and methods going into far greater detail of the individual methods used than some of the pure method focused reviews.

Although most of the machine learning methods used supervised techniques, these all require large quantities of annotated data, with both volume and the annotation quality having direct influence on the resulting accuracy. Annotating a dataset is a time intensive, and often expensive process as it requires domain specific knowledge [21, 44]. The general lack of availability of health specific trained or labelled data has implications for the accuracy that can be achieved. Zunic [74] suggests that increased use of shared datasets could increase the use of deep learning methods, improving the performance levels, as well enabling comparison between methods. However the use of complex deep learning methods requires a trade-off between the computational cost involved and the performance levels that can be achieved [58].

### RQ4: knowledge gaps

The strongest message from these reviews was confirming how much more work there is to do in this emerging area. Knowledge gaps, defined areas for future research, as well as limitations with existing studies were identified in all the included papers. Despite the increase in interest in this area in the last few years, a recent review that looked at Covid-19 related social media found that there was a lack of studies both into the application of machine learning on the data and into its use for real time surveillance [80]. The lack of systematic testing of methods and results impacts on the credibility of the findings. The importance of reproducibility is a current issue in healthcare research, so future work in this area should make clear what methods are used [81].

From the literature to date, there seemed to be little evidence of this data source being used to assess or evaluate the patient perspective of the effectiveness of a treatment, intervention, or service, other than for detecting adverse events or reactions. Given that so much is still unknown about the relationships between patient characteristics, environment and disease, patterns of symptoms, behaviours or effects that can be extracted from SGOPE may give new insights that can be used to improve outcomes [82].

Although many of the reviews acknowledged that sharing knowledge between online users was one of the big factors in online health information use [20] only one of the reviews focused on how important this was to those with long term conditions [26]. Very few of the studies had explored or compared unsupervised methods to identify the themes being discussed.

Few reviews looked at identifying any form of inferred or perceived causal inference from the social media posts. Dictionary-based systems that can match explicit interventions and symptoms within defined units of text can be a simple and very effective way of identifying potential relationships [83]. Determining that a possible relationship exists is however, different to determining what the actual effect is, especially if as a retrospective event. Very few of the review studies look beyond the co-occurrences of named entities to indicate a possible relationship between items. There has been less focus on assessing causality to identify true drug-ADE pairs [44, 51]. One suggestion is that the low quality of the information precludes the evaluation of causal links [31, 51], although the quality of the posts in terms of completeness varies widely between sites [51]. Identifying temporal data to sequence events could help distinguish true causal links [44], as will the continued working on the more complex tasks of lexical semantics, coreference resolution and discourse analysis [55].

### Strengths and limitations

Using an umbrella scoping review approach summarises the current state of the art of this fast-moving field. One of the strengths of this method is that although some of the individual studies may have been included in multiple reviews, each review paper has had different research questions, thus generating a range of different perspectives on any such papers. Seven databases were searched, together with grey literature and reference lists. It is however subject to the usual limitations of the keyword-based searches, in that it is possible that some relevant literature may have been missed. Searches were also limited to those in English. However this was mitigated by the deliberately broad inclusion / exclusion criteria which were intended to ensure that as many as possible relevant reviews were included in the final analysis.

## Conclusion

SGOPE data remains an underused resource in healthcare. It has the potential to increase knowledge of many different aspects of healthcare and as such has a multitude of potential uses. Despite the raft of suggestions for future research and methodological development that is needed, the consensus from the included reviews in this study is that SGOPE is a data source capable of offering considerable benefit to healthcare researchers and providers, and that NLP will become an important methodological tool within health research.

## Supplementary Information

**Additional file 1.**

## Data Availability

Not applicable.

## Abbreviations

ADR: Adverse Drug Reaction
ADE: Adverse Drug Event
API: Application Programming Interface
EHR: Electronic Health Record
OSN: Online Social Networks
NER: Named Entity Recognition
NLP: Natural Language Processing
SM: Social Media
SoMe: Social Media
UMLS: Unified Medical Language System
