# Supplementary material for: Spontaneously generated online patient experience data - how and why is it being used in health research: an umbrella scoping review
Source: BMC Med Res Methodol. 2022 May 14;22:139. doi: 10.1186/s12874-022-01610-z (PMC9106384; doi:10.1186/s12874-022-01610-z)
Supplement: Supplementary file 1 — Additional file 1. [file 12874_2022_1610_MOESM1_ESM.docx]

**PubMed search string:**

Search: ((((social media OR "online space" OR "social network*"OR "online network*" OR blog* OR microblog OR "discussion forum*" OR "online community" OR facebook OR twitter OR patientslikeme OR askapatient OR healthunlocked OR webmd OR reddit OR "health forum*" OR "patient forum*" OR "patient communit*" OR "online patient communit*" OR "message board" OR "online health communit*") AND ((review[Filter] OR systematicreview[Filter]) AND (humans[Filter]) AND (english[Filter]))) and ((analysis or "data mining" or "text mining" or infoveillance or crowdsourc* or "crowd-sourc*" or "social listening" or dataveillance or "social media listening" or "social media mining" or "signal detection" or "content analysis") AND ((review[Filter] OR systematicreview[Filter]) AND (humans[Filter]) AND (english[Filter])))) AND ((review[Filter] OR systematicreview[Filter]) AND (humans[Filter]) AND (english[Filter]))) and (((("natural language processing" or NLP or "natural language understanding" or NLU or "machine learning" or "artificial intelligence" or AI or 'deep learning' or reinforcement learning' or 'transfer learning' or 'neural network' or algorithm* or 'corpus linguistics' or network analysis' or 'sentiment analysis' or classif* or cluster* or 'topic model*' or semantic)) AND ((review[Filter] OR systematicreview[Filter]) AND (humans[Filter]) AND (english[Filter]))) OR (('word embedding' or supervised or unsupervised or 'semi*supervised' or annotation or dictionar* or 'rule*based' or SVM or 'decision tree' or 'knowledge graph' or CRF or 'dictionary based' or 'rules based' or 'Latent Dirichlet' or LDA) AND ((review[Filter] OR systematicreview[Filter]) AND (humans[Filter]) AND (english[Filter]))) OR ((GATE or CTakes or MetaMap or word2vec or doc2vec or GloVe or XLNet or BRAT or WebAnno or tagtog or LightTag or StanfordNLP or nltk or spacy or gensim) AND ((review[Filter] OR systematicreview[Filter]) AND (humans[Filter]) AND (english[Filter]))) OR ((UMLS or SNOMED or RxNorm or SIDER or MedDRA or lexicon or ontology) AND ((review[Filter] OR systematicreview[Filter]) AND (humans[Filter]) AND (english[Filter])))) AND ((review[Filter] OR systematicreview[Filter]) AND (humans[Filter]) AND (english[Filter]))) Filters: Review, Systematic Review, Humans, English, from 2020 – 2021

Web of Science search

| 1 | TS=(health or disease or illness) | 7,150.246 |
| --- | --- | --- |
| 2 | TS=("social media" OR "online space" OR "social network*"OR "online network*" OR blog* OR microblog OR "discussion forum*" OR "online community" OR facebook OR twitter OR patientslikeme OR askapatient OR healthunlocked OR webmd OR reddit OR "health forum*" OR "patient forum*" OR "patient communit*" OR "online patient communit*" OR "message board" OR "online health communit*" ) | 211,199 |
| 3 | 1 AND 2 | 36,887 |
| 4 | TS=("social media analysis" or "data mining" or "text mining" or infoveillance or crowdsourc* or "crowd-sourc*" or "social listening" or dataveillance or "social media listening" or "social media mining" or "signal detection" or "content analysis" ) | 204,274 |
| 5 | 3 AND 4 | 2,656 |
| 6 | ALL=(symptom* or "side effect" or "side-effect" or "adverse drug reaction" or ADR or effectiv* or outcome or "quality of life" or qol or HRqol or impact or causa* or "causa* link" or" causa* relationship" or drug or intervention or pharmocovigilance) | 12,891,431 |
| 7 | TS=(analysis or "data mining" or "text mining" or infoveillance or crowdsourc* or "crowd-sourc*" or "social listening" or dataveillance or "social media listening" or "social media mining" or "signal detection" or "content analysis" ) | 11,173,390 |
| 8 | TS=("patient experience" or "patient report*" or "self report*" or "patient generated" or "user generated" or post* or message or thread or conversation or EPAT or UGC or "patient authored" or "knowledge sharing") | 3,969,184 |
| 9 | 7 and 3 | 15.405 |
| 10 | 8 or 6 | 15,290,232 |
| 11 | 10 and 5 | 2,039 |
| 12 | 10 and 9 | 11,256 |
| 13 | 10 and 5 : Language = English, Document Type = Review | 97 |
| 14 | 10 and 9 : Language = English, Document Type = Review | 671 |
| 15 | (TS=("natural language processing" or NLP or "natural language understanding" or NLU or "machine learning" or "artificial intelligence" or AI or 'deep learning' or reinforcement learning' or 'transfer learning' or 'neural network' or algorithm* or 'corpus linguistics' or network analysis' or 'sentiment analysis' or classif* or cluster* or 'topic model*' or semantic)) AND LANGUAGE: (English) | 5,503,325 |
| 16 | (TS=('word embedding' or supervised or unsupervised or 'semi*supervised' or annotation or dictionar* or 'rule*based' or SVM or 'decision tree' or 'knowledge graph' or CRF or 'dictionary based' or 'rules based' or 'Latent Dirichlet' or LDA)) AND LANGUAGE: (English) | 664,775 |
| 17 | (TS= (GATE or CTakes or MetaMap or word2vec or doc2vec or GloVe or XLNet or BRAT or WebAnno or tagtog or LightTag or StanfordNLP or nltk or spacy or gensim)) AND LANGUAGE: (English) | 338,118 |
| 18 | (TS=(UMLS or SNOMED or RxNorm or SIDER or MedDRA or lexicon or ontology)) AND LANGUAGE: (English) | 108,863 |
| 19 | 18 or 17 or 16 or 15 | 6,151,579 |
| 20 | 19 and 11 | 1,163 |
| 21 | 19 and 12 | 7,304 |
| 22 | 19 and 11: Language = English, Document Type = Review | 63 |

Medline, Embase, PsychInfo

| 1 | ("social media" or "online space" or "social network*OR online network*" or blog* or microblog or "discussion forum*" or "online community" or facebook or twitter or patientslikeme or askapatient or healthunlocked or webmd or reddit or "health forum*" or "patient forum*" or "patient communit*" or "online patient communit*" or "message board" or "online health communit*").mp. [mp=title, abstract, original title, name of substance word, subject heading word, floating sub-heading word, keyword heading word, organism supplementary concept word, protocol supplementary concept word, rare disease supplementary concept word, unique identifier, synonyms] |  |
| --- | --- | --- |
| 2 | ("social media analysis" or "data mining" or "text mining" or infoveillance or crowdsourc* or "crowd-sourc*" or "social listening" or dataveillance or "social media listening" or "social media mining" or "signal detection" or "content analysis").mp. [mp=title, abstract, original title, name of substance word, subject heading word, floating sub-heading word, keyword heading word, organism supplementary concept word, protocol supplementary concept word, rare disease supplementary concept word, unique identifier, synonyms] |  |
| 3 | 1 and 2 |  |
| 4 | limit 3 to (english language and "review articles" and humans) |  |
| 5 | ("patient experience" or "patient report*" or "self report*" or "patient generated" or "user generated" or post* or message or thread or conversation or EPAT or UGC or "patient authored" or "knowledge sharing").mp. [mp=title, abstract, original title, name of substance word, subject heading word, floating sub-heading word, keyword heading word, organism supplementary concept word, protocol supplementary concept word, rare disease supplementary concept word, unique identifier, synonyms] |  |
| 6 | (symptom* or "side effect" or "side-effect" or "adverse drug reaction" or ADR or effectiv* or outcome or "quality of life" or qol or HRqol or impact or causa* or "causa* link" or " causa* relationship" or drug or intervention or pharmocovigilance).mp. [mp=title, abstract, original title, name of substance word, subject heading word, floating sub-heading word, keyword heading word, organism supplementary concept word, protocol supplementary concept word, rare disease supplementary concept word, unique identifier, synonyms] |  |
| 7 | 5 or 6 |  |
| 8 | 3 and 7 |  |
| 9 | limit 8 to (english language and "review articles" and humans) |  |
| 10 | ("natural language processing" or NLP or "natural language understanding" or NLU or "machine learning" or "artificial intelligence" or AI or 'deep learning' or reinforcement learning' or 'transfer learning' or 'neural network' or algorithm* or 'corpus linguistics' or network analysis' or 'sentiment analysis' or classif* or cluster* or 'topic model*' or semantic).mp. [mp=title, abstract, original title, name of substance word, subject heading word, floating sub-heading word, keyword heading word, organism supplementary concept word, protocol supplementary concept word, rare disease supplementary concept word, unique identifier, synonyms] |  |
| 11 | ('word embedding' or supervised or unsupervised or 'semi*supervised' or annotation or dictionar* or 'rule*based' or SVM or 'decision tree' or 'knowledge graph' or CRF or 'dictionary based' or 'rules based' or 'Latent Dirichlet' or LDA).mp. [mp=title, abstract, original title, name of substance word, subject heading word, floating sub-heading word, keyword heading word, organism supplementary concept word, protocol supplementary concept word, rare disease supplementary concept word, unique identifier, synonyms] |  |
| 12 | (GATE or CTakes or MetaMap or word2vec or doc2vec or GloVe or XLNet or BRAT or WebAnno or tagtog or LightTag or StanfordNLP or nltk or spacy or gensim).mp. [mp=title, abstract, original title, name of substance word, subject heading word, floating sub-heading word, keyword heading word, organism supplementary concept word, protocol supplementary concept word, rare disease supplementary concept word, unique identifier, synonyms] |  |
| 13 | (UMLS or SNOMED or RxNorm or SIDER or MedDRA or lexicon or ontology).mp. [mp=title, abstract, original title, name of substance word, subject heading word, floating sub-heading word, keyword heading word, organism supplementary concept word, protocol supplementary concept word, rare disease supplementary concept word, unique identifier, synonyms] |  |
| 14 | 10 or 11 or 12 or 13 |  |
| 15 | 8 and 14 |  |
| 16 | limit 15 to (english language and humans) |  |
| 17 | limit 16 to "review articles" |  |
| 18 | limit 8 to (english language and humans) |  |
| 19 | (analysis or "data mining" or "text mining" or infoveillance or crowdsourc* or "crowd-sourc*" or "social listening" or dataveillance or "social media listening" or "social media mining" or "signal detection" or "content analysis").mp. [mp=title, abstract, original title, name of substance word, subject heading word, floating sub-heading word, keyword heading word, organism supplementary concept word, protocol supplementary concept word, rare disease supplementary concept word, unique identifier, synonyms] |  |
| 20 | 1 and 19 |  |
| 21 | limit 20 to (english language and humans) |  |
| 22 | limit 21 to "review articles" |  |
| 23 | 7 and 21 |  |
| 24 | limit 23 to (english language and "review articles" and humans) |  |
| 25 | 14 and 23 |  |
| 26 | limit 25 to (english language and "review articles" and humans) |  |
| 27 | limit 26 to (english language and humans) |  |
| 28 | limit 25 to (english language and humans) |  |
| 29 | 3 and 14 |  |
| 30 | limit 29 to (english language and humans) |  |
| 31 | limit 30 to "review articles" |  |

**ACM**

"query": { Keyword:(social media) AND Keyword:(health) }

"filter": { Publication Date: (01/01/2015 TO 01/31/2021), ACM Content: DL }

**IEEE Xplore**

(("Mesh_Terms":social media) OR "Author Keywords":social media), health
